# Forging a sustainable sky: Unveiling the pillars of aviation e-fuel production for carbon emission circularity

**DOI:** 10.1016/j.isci.2024.109154

**Published:** 2024-02-22

**Authors:** Mihrimah Ozkan, Anvaya B. Narappa, Thrayesh Namboodiri, Yijian Chai, Matheshwaran Babu, Joan S.E. Jennings, Yingfan Gao, Sameeha Tasneem, Jason Lam, Kamal R. Talluri, Ruoxu Shang, Cengiz S. Ozkan, Jordyn M. Watkins

**Affiliations:** 1 Department of Electrical and Computer Engineering, University of California, Riverside, Riverside, CA, USA; 2 Department of Computer Science, University of California, Riverside, Riverside, CA, USA; 3 Department of Mechanical Engineering, University of California, Riverside, Riverside, CA, USA; 4 Materials Science and Engineering Program, University of California, Riverside, Riverside, CA, USA

**Keywords:** Electrochemical energy production, Biofuel, Fuel technology, Energy sustainability

## Abstract

In 2021, airplanes consumed nearly 250 million tons of fuel, equivalent to almost 10.75 exajoules. Anticipated growth in air travel suggests increasing fuel consumption. In January 2022, demand surged by 82.3%, as per the International Air Transport Association. In tackling aviation emissions, governments promote synthetic e-fuels to cut carbon. Sustainable aviation fuel (SAF) production increased from 1.9 million to 15.8 million gallons in six years. Although cost of kerosene produced with carbon dioxide from direct air capture (DAC) is several times higher than the cost of conventional jet fuel, its projected production cost is expected to decrease from $104–$124/MWh in 2030 to $60–$69/MWh in 2050. Advances in DAC technology, decreasing cost of renewable electricity, and improvements in FT technology are reasons to believe that the cost of e-kerosene will decline. This review describes major e-kerosene synthesis methods, incorporating DAC, hydrogen from water electrolysis, and hydrocarbon synthesis via the Fischer-Tropsch process. The importance of integrating e-fuel production with renewable energy sources and sustainable feedstock utilization cannot be overstated in achieving carbon emission circularity. The paper explores the concept of power-to-liquid (PtL) pathways, where renewable energy is used to convert renewable feedstocks into e-fuels. In addition to these technological improvements, carbon pricing, government subsidies, and public procurement are several policy initiatives that could help to reduce the cost of e-kerosene. Our review provides a comprehensive guide to the production pathways, technological advancements, and carbon emission circularity aspects of aviation e-fuels. It will provide a valuable resource for researchers, policymakers, industry stakeholders, and the general public interested in transitioning to a sustainable aviation industry.

## Graphical abstract

**Figure:**
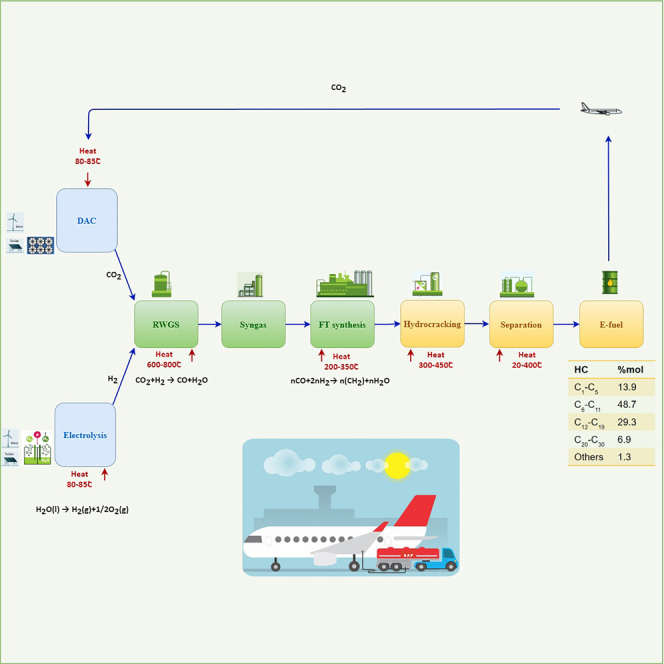

## Introduction

Aviation emissions in 2021 amounted to approximately 720 million tons of CO_2_, accounting for about 3%–4% of global-energy-related CO_2_ emissions.^1^^,^^2^ Although this percentage may seem small, it is a growing concern due to the increasing number of people traveling by air every year. In 2018, only 11% of people took to the skies, but this number is rising rapidly, and air-travel-related emissions are growing faster than road-, railway-, or shipping-related emissions.^3^ Reducing CO_2_ emissions in aviation is challenging because airplanes require high energy-density propulsion to transport heavy weights over long distances. Standard jet fuels such as kerosene have a much higher energy density than common energy sources, such as the Li-ion battery (12,000 Wh/kg versus 100–265 Wh/kg, respectively).^4^ This means roughly 50 kg of battery storage would be required to generate an energy equivalent to 1 kg of kerosene, which is just an unreasonable amount of weight for an airplane to carry. Advancements in aviation infrastructure have been slow, and current aircraft designs may require significant modification to employ upcoming alternatives such as hydrogen fuels. The need for major physical design alterations constitutes a large entry barrier for alternative fuels as such modifications are exceedingly costly due to safety certification requirements. Fortunately, drop-in fuels such as alternative sustainable aviation fuels (SAFs) are available, which are synthetic and interchangeable substitutes that do not require any modifications to the aircraft infrastructure.^5^

SAF is created from renewable feedstocks such as municipal waste, agricultural residues, and waste lipids. Since 2011, it has been used to fuel more than 484,000 commercial flights.^6^ When SAF is blended with conventional jet fuel, Jet A1, it has been shown to significantly reduce sulfur oxides (SO_x_) and particulate matter (PM) emissions while generally reducing unburned hydrocarbon (UHC) and carbon monoxide (CO) emissions. Nitrogen oxide (NO_x_) emissions are minimally reduced or unaffected. In addition, SAF has been found to reduce greenhouse gas (GHG) emissions by 85%–100% compared with fossil-based jet fuels, according to a life-cycle analysis. By blending as little as 50% SAF with commercial fuels, significant emissions reduction can be achieved, and it is expected that up to 100% SAF could be used in the future as more fuel stock becomes available.^7^ By using SAF in aviation, the industry is taking an active step toward a net-zero scenario. SAF use in all aviation fuels is projected to reach nearly 10% by 2030.

McKinsey reports enough feedstock to produce over 400 million tons of SAF. However, the cost of SAF is still a limiting factor for its widespread use, and it depends mainly on the costs of hydrogen and CO_2_ feedstocks. In this review, authors mainly focus on e-kerosene, which is relatively expensive due to CO2 and green hydrogen sources but has a much better carbon balance than alternative biofuels. The production cost of e-kerosene is dominated by hydrogen, which accounts for 66%–83% of the cost, followed by the cost of CO_2_ capture.^8^ The cost of renewable energy needed during production is the main factor determining the costs of renewable hydrogen and CO_2_ from direct air capture (DAC).

**Figure 1:**
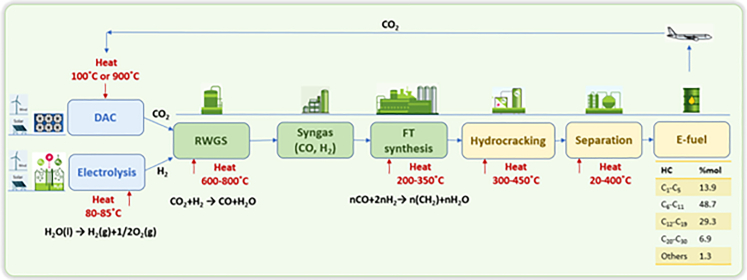
The e-fuel production involves producing hydrogen through water electrolysis and sourcing CO_2_ via direct air capture DAC technology extracts CO_2_ directly from the atmosphere to create aviation e-fuels, which form a closed-loop system. This approach aligns with the circular economy concept, reduces aviation’s carbon footprint, and contributes to the fight against climate change. DAC, direct air capture; RWGS, the reverse water gas shift; FT, Fischer-Tropsch; HC, hydrocarbon.

The process for producing e-kerosene (Figure 1) involves hydrogen production through the electrolysis of water and CO_2_ obtained through DAC. FT synthesis is used to convert hydrocarbons into jet fuel. The captured CO_2_ is converted into syngas (CO and H_2_) through RWGS. All these processes, except for the RWGS reactor, are mature and operate at an industrial scale. Several electrolysis technologies for hydrogen production include alkaline electrolysis, proton exchange membrane electrolysis, and solid oxide electrolysis.^9^ These technologies differ in efficiency, investment and maintenance costs, durability and lifespan, capacity, and flexibility. CO_2_ can be obtained through DAC using physical or chemical methods. Adsorption technologies use liquid or solid adsorbents, whereas chemical adsorption uses alkanolamine or potassium carbonate. DAC technologies are more expensive as they require higher energy inputs and larger volumes of air to be processed.^10^^,^^11^^,^^12^^,^^13^ Synthesis technologies such as Fischer-Tropsch (FT) require CO, which necessitates the conversion of CO_2_ into CO, which may be done in a separate reactor. However, direct one-step conversion of CO_2_ and H_2_ is being investigated by companies such as Air Company and Carbon Recycling International, which use this process to produce methanol. Methanol is then converted into longer-chain hydrocarbons.

The FT process for synthesizing aviation fuels was certified by ASTM in 2009, making it possible for fuels produced through this process to enter the market immediately. Because the syngas used in e-fuel production are expected to be relatively pure, limited syngas cleanup may be required. However, one of the main challenges for this technology is the availability of sufficient renewable energy and the high cost of e-fuel production. If renewable energy were directed toward liquid fuel production, it would face direct competition with other initiatives to decarbonize electricity and transportation, including the push for more electric vehicles worldwide.

This review discusses various aspects of e-fuel production, including carbon sources from DAC and hydrogen sources from electrolysis. It also covers hydrocarbon synthesis using the FT process, energy demands of individual processes, costs, investment, policy requirements, and the gaps between each. The demand for the area and a comparison between biomass feedstock and DAC carbon sources are explained, and the short-term and long-term actions required to reduce the cost of e-fuels and ramp up production scale to meet demand are discussed in detail.

## Overview of aviation e-fuels

Different types of e-fuels can be used to replace traditional fuels in aviation. These e-fuels, also known as power-to-liquid (PtL) synthetic fuels, are an innovative type of SAF that offers a promising solution for powering future aircraft. SAFs are unique because they are made from renewable biomass or waste-based feedstock, which inherently have a lower life-cycle carbon intensity than conventional petroleum-based fuel. Various feedstocks can be used for SAF production, such as oilseed plants, energy grasses, agricultural and forestry residue, organic municipal solid waste, fats, oils, and greases (FOGs) from cooking waste and meat production, algae, and industrial carbon monoxide waste gas. FOGs are the most commonly used feedstock for SAF despite their limited supply. SAFs are classified based on their carbon feedstock into hydroprocessed esters and fatty acids (HEFA), Fischer-Tropsch synthetic paraffinic kerosene (FT-SPK), alcohol-to-jet (AtJ), and pyrolysis oil-to-jet (PO-TJ).^14^

Among e-fuels, e-kerosene (Jet A) is the most feasible and promising option. E-kerosene is produced by mixing hydrogen (H_2_) and carbon dioxide (CO_2_), as implemented by the companies Synkero and Johnson Matthey. Renewable electricity (known as “green hydrogen”) and DAC technologies are used to obtain hydrogen and carbon dioxide sustainably. When renewable electricity is used to electrolyze water, the production of e-kerosene could potentially result in close-to-zero greenhouse gas (GHG) emissions. Using e-kerosene significantly reduces CO_2_ emissions, with only minimal residual emissions. Additionally, the tech outlook for e-kerosene seems promising, as improvements in renewable electricity, green hydrogen production, and DAC technology are expected. The International Council on Clean Transportation has evaluated the cost of e-kerosene in the United States and Europe, both in the present and projected future.^15^ Reports suggest that e-kerosene could be priced on par with taxed fossil kerosene by the 2030s, making it a more attractive option from both cost and climate perspectives.^16^^,^^17^^,^^18^

Comparing e-fuels for aviation requires balancing volumetric and gravimetric energy densities and their aviation readiness. Hydrogen-based e-fuels have high volumetric energy densities but face storage and infrastructure challenges. Synthetic hydrocarbons have competitive gravimetric energy densities, are compatible with existing aviation infrastructure, and are a practical solution for transitioning to SAFs. E-kerosene has a volumetric density comparable with diesel fuels and can be considered a drop-in fuel with no infrastructure modification. However, hydrogen with a low boiling point of −252°C requires modification to existing infrastructure.

**Figure 2:**
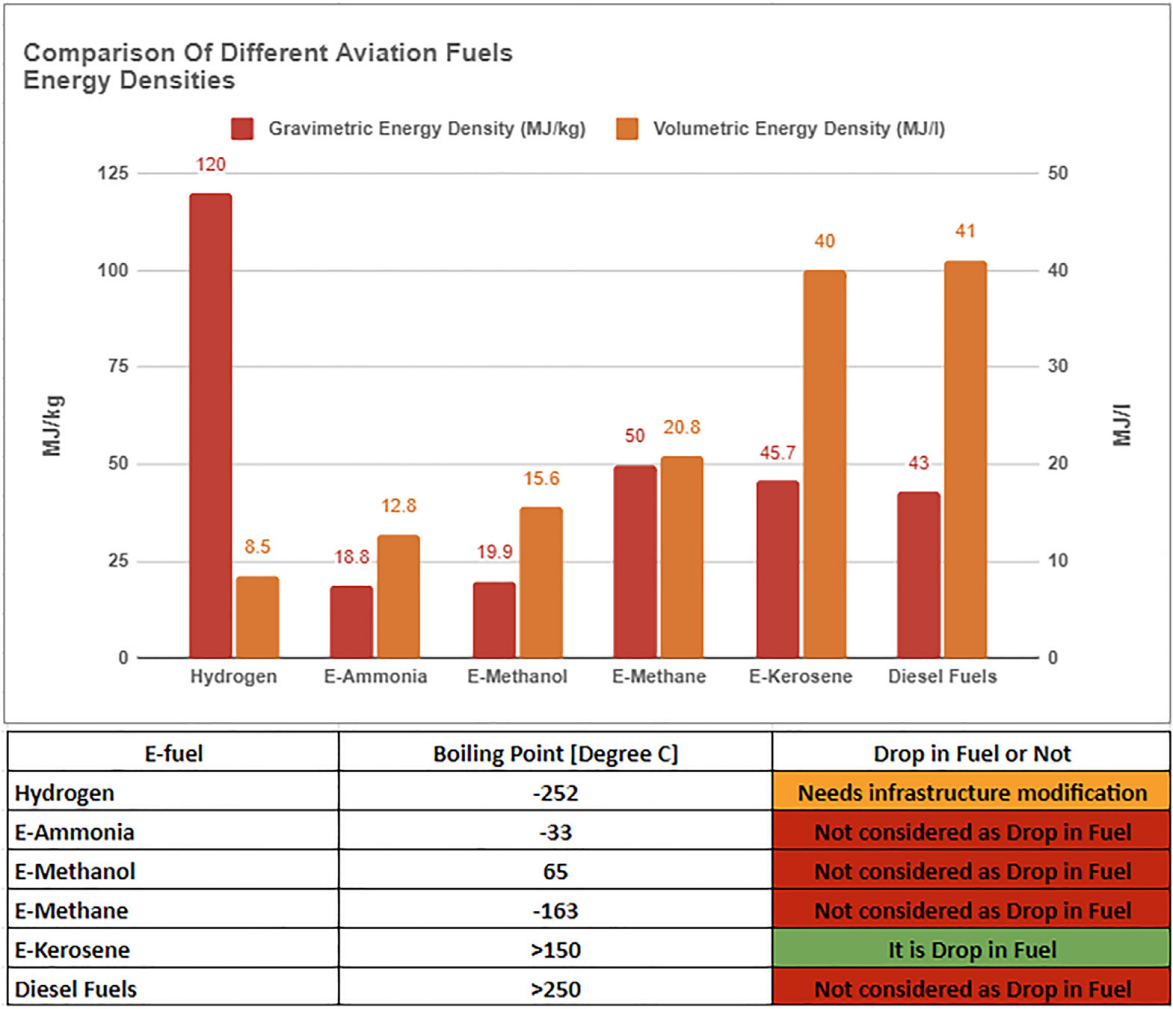
Comparison of various e-fuels, highlighting their volumetric and gravimetric energy densities and readiness to be usable in aviation

In Figure 2, a comparison of different types of e-fuels based on their volumetric and gravimetric energy density is presented. Diesel fuels have the highest volumetric density, whereas hydrogen has the highest gravimetric density. However, e-kerosene has a volumetric energy density comparable with diesel fuel. From this comparison, it can be inferred that most e-fuels are not drop-in fuels and require some modification before use. Hydrogen requires infrastructure modification, whereas e-kerosene can be used without any hassle. E-kerosene is a type of SAF compatible with conventional fuels and combustion engines. It combines carbon dioxide (CO_2_) and hydrogen from water electrolysis.^15^

The European Commission has recently proposed a mandate for SAFs, which would require a 5% share of aviation fuel to be SAF, with a sub-target of 0.7% for e-kerosene, by 2030. The proposed SAF target increases to 63%, with a minimum of 28% e-kerosene by 2050.^15^

The estimated production cost of e-kerosene in the EU was $12.40 per gallon in 2020, but it is expected to decrease to $6.70 per gallon by 2050. However, e-kerosene costs in the EU are higher than in other regions due to more expensive renewable electricity.

Finally, it is crucial to consider the current and planned e-fuel production status, as it is an essential factor in achieving net zero emissions. In the next section, the status of current and planned e-fuel production is discussed.

### A comparative analysis of standard and synthetic aviation e-fuels—assessing their climate impacts

**Figure 3:**
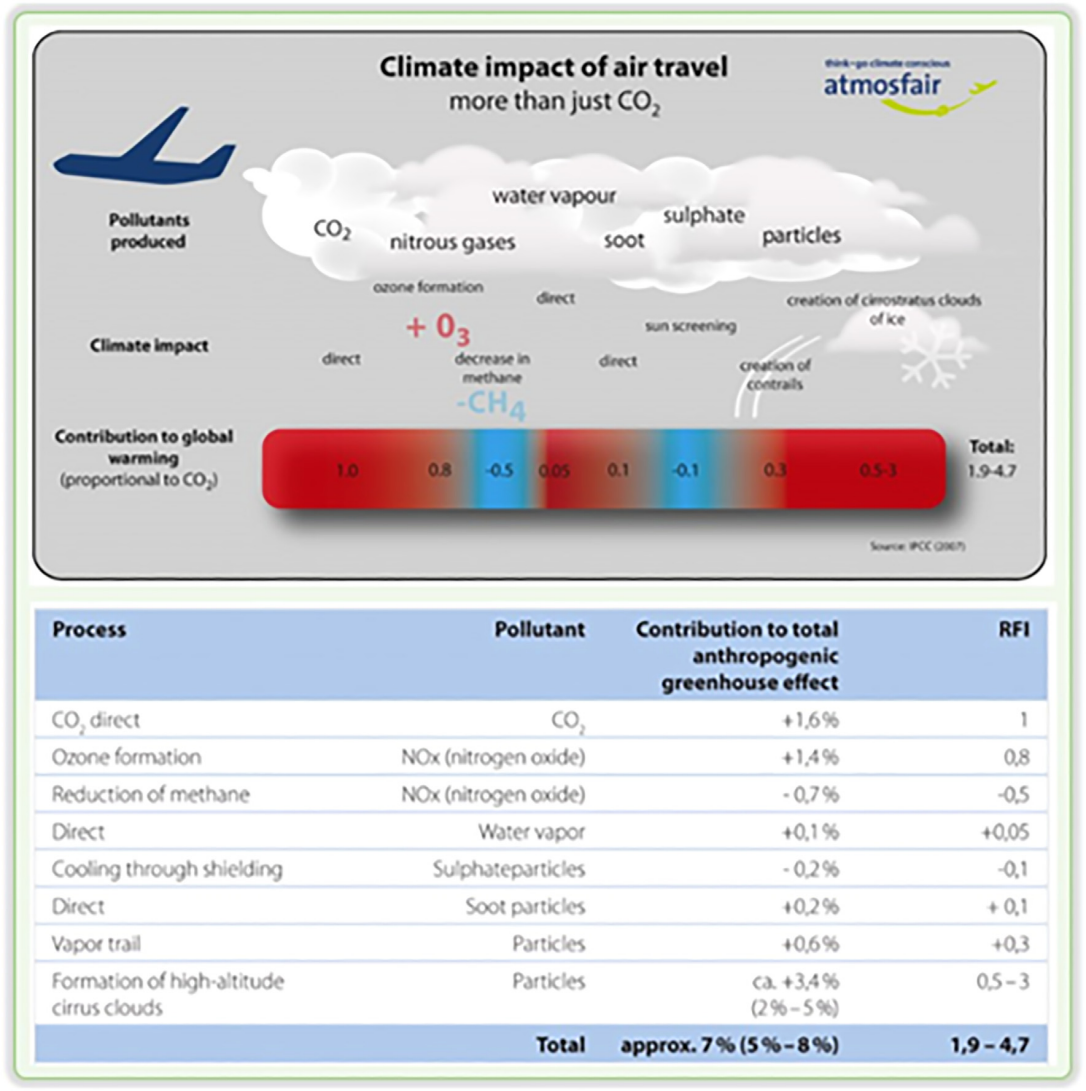
Climate impact of air travel

There are three key processes by which aircraft emissions cause atmospheric changes: (1) direct emission of substances such as CO_2_ or water vapor (H_2_O); (2) emission of chemical species that produce or destroy radiatively active substances, including NO_x_, which affects O_3_ concentration; and (3) emission of substances that induce the formation of aerosol particles or cause changes in natural clouds, such as the formation of contrails. Figure 3 summarizes these processes and the resulting substances during flight, and the following section addresses these three processes for conventional and emerging synthetic fuels, as summarized in Table 1.

**Table 1:** Assessing environmental impacts of alternative jet fuels from different feedstock

Alternative jet fuel	Feedstock	Process	Emission impact
SIP (synthesized isoparaffins)	Biomass gas or natural gas	Isoparaffins are prepared from gases through a synthesis process	Reduced major gaseous emissions Produce NO_x_ Produce PM emission
HEFA (hydroprocessed esters and fatty acids)	Vegetable oils, fats, and other biomass	Hydrolysis and deoxygenation reactions	Reduced major gaseous emissions Produce NO_x_ Reduced PM emission
BtL (biomass to liquid)	Alcohol (CH_3_OH) CO_2_, hydrogen	Produced by thermochemical (gasification) or biochemical (fermentation) methods	Reduced major gaseous emissions Produce NO_x_ Reduced PM emission
ATJ (alcohol to jet)	Biomass (wood, crop residues, biomass waste, etc.)	Dehydration, alkyd reaction, and etherification	Reduced major gaseous emissions Produce NO_x_ Reduced PM emission
E-kerosene	Hydrogen, CO_2_, with electricity from renewable sources Or solar energy	Convert energy to hydrogen, then combine with CO_2_ to make E-kerosene	Slightly reduced major gaseous emissions Reduce NO_x_ emission Slightly reduced PM emission

Adapted from ref.^19^

Compared with traditional fossil fuels like Jet A1, e-fuels are much cleaner and can significantly reduce CO_2_ emissions. This is partly because they are produced using renewable energy sources such as solar, wind, and hydroelectric power, resulting in minimal carbon emissions during production.^20^ E-fuels produce fewer soot and ice particles, which not only reduces energy deposition in the atmosphere but also has a positive impact on reducing the greenhouse effect.^21^ According to NASA’s AAFEX results, synthetic fuels burn cleaner than conventional fuels, producing significantly less PM and HAPS.^22^ Therefore, reducing CO_2_ contributions can be achieved by switching to renewable energy sources such as green hydrogen and synthetic fuel.

To quantify the effect of aviation on climate change, the Radiative Forcing Index (RFI), which measures the ratio of total radiative forcing to that from CO_2_ emissions alone, is used to determine the extent of aircraft-induced climate change other than that caused by the release of fossil carbon alone. Like any other individual sector, aircraft radiative forcing contributes only a small fraction to the overall anthropogenic climate forcing, which is currently about 4%. According to the F-type and E-type scenarios presented in this reference, this fraction could reach 3%–7% and 10%–15% by 2050. The most significant areas of scientific uncertainty when predicting aircraft-induced climate effects and RFI values are persistent contrails, increases in tropospheric ozone and the resultant changes in methane, potential particle impacts on clouds, and changes in water vapor and ozone in the lower stratosphere, particularly during supersonic flights.^23^

The aircraft-induced radiative forcing index estimates the impact of aviation on climate change. The following are the estimated values for different factors: CO_2_, +0.018 Wm^−2^; NO_x_, +0.023 Wm^−2^ (via ozone changes) and −0.014 Wm^−2^ (via methane changes); contrails, +0.02 Wm^−2^; stratospheric H_2_O, +0.002 Wm^−2^; sulfate aerosol (direct effect), −0.003 Wm^−2^; and black carbon aerosol (soot), +0.003 Wm^−2^ (as shown in Figure 3). Changes in natural cirrus clouds caused by aircraft may result in a negligible or potentially large radiative forcing effect, with an estimate ranging between 0 and 0.04 Wm^−2^ (Figure 3).

The RFI (radiative forcing index) 2050, including high-speed civil transport (HSCT) and supersonic aviation, ranges from 2.2 to 3.4. The primary cause of this increase is the effect of stratospheric water vapor. Therefore, aircraft-induced climate change with RFI greater than 1 indicates the need for a more thorough climate assessment for this sector.

Three types of atmospheric processes result from aircraft. The first is the direct emission of active substances, such as CO_2_ or water vapor. The second is the emission of chemical species that produce or destroy radiatively active substances, like NO_x_, which modifies O_3_ concentration. The third type of process is the emission of substances that trigger the generation of aerosol particles or lead to changes in natural clouds. The Radiative Forcing Index (RFI) measures the importance of aircraft-induced climate change, other than that from the release of fossil carbon alone. It is calculated as the ratio of total radiative forcing to that from CO_2_ emissions alone.^24^

**Figure 4:**
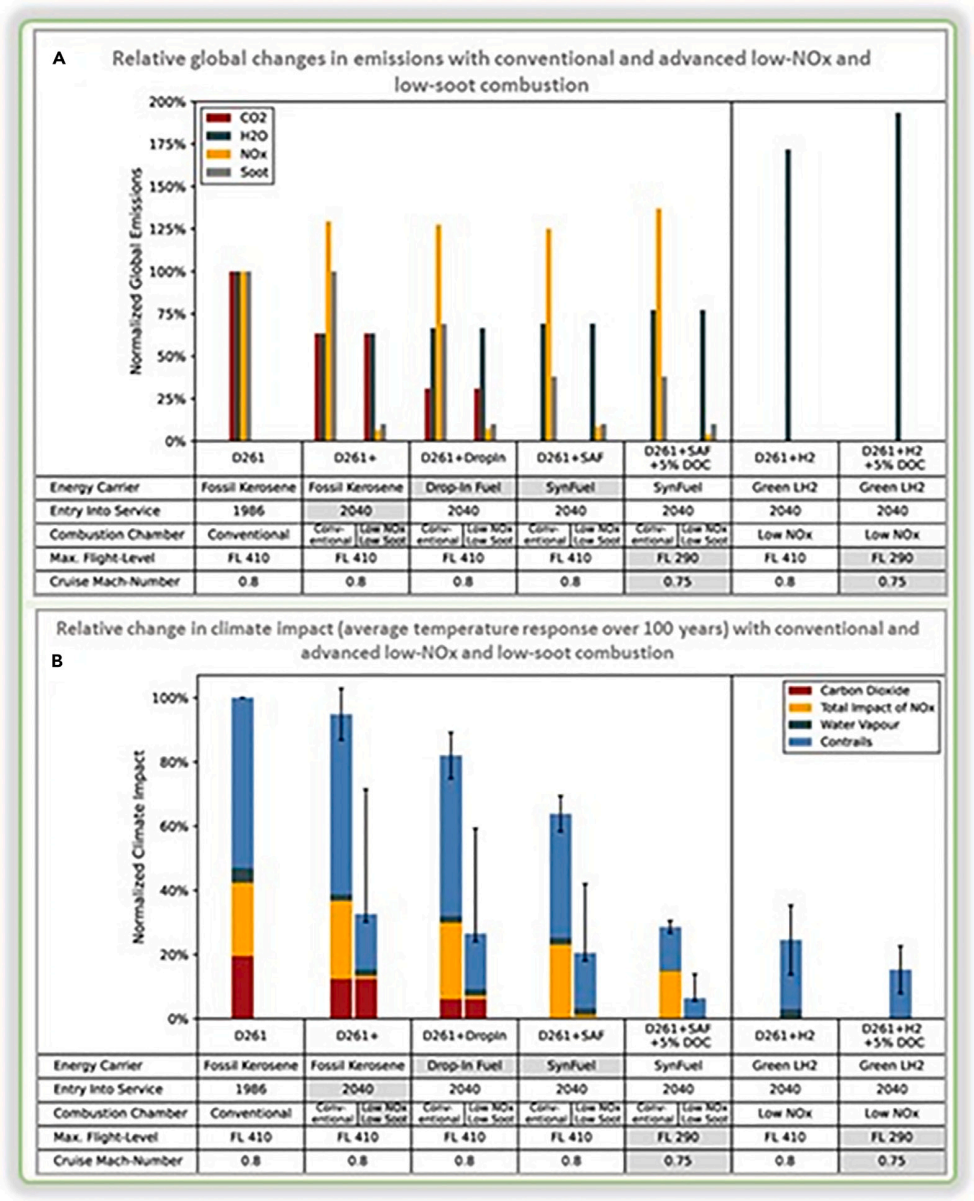
Emission and climate impact of conventional, advanced low-NO_x_ and low-soot combustion (A) Relative global changes in emissions with conventional and advanced low-NO_x_ and low-soot combustion. The gray cells indicate the changes in the new scenario compared with the preceding neighbor.^25^ (B) Relative change in climate impact (ATR100) with conventional and advanced low-NO_x_ and low-soot combustion. The gray cells indicate the changes in the new scenario compared with the preceding neighbor. The black whisker lines visualize the uncertainties. Both the drop-in blend and the PtL synthetic kerosene utilize direct air capturing (DAC) as the carbon source and green hydrogen. D261: Reference of B767–300, a medium and large twin-engine jet airliner produced by Boeing, one of the leading civil aviation models. D261+: predict the improvement of the fossil fuel, the baseline of service in 2040. D261+ Dropping: the D261+ with the use of a 50% drop-in mix. D261+ SAF: the D261+ with the use of 100% synthetic kerosene. Conducted the attitude constraint and cruise Mach number trade. D261 + H2: the D261+ with the use of 100% green liquid hydrogen. Conducted the attitude constraint and cruise Mach number trade. DOC: direct operating costs. Applying a flight-level constraint of 290 and a cruise Mach number of 0.75, causing 5% higher average direct operating costs (DOC). Max-flight level: flight level restrictions. FH290 means the flight level restriction of 290. LH2: liquid hydrogen.

Figure 4A examines the relative change in global emissions using conventional and advanced low-NO_x_ and low-smoke combustion technologies. The gray cell indicates the change of the new scheme compared with the previous scheme, that is, the impact on global emissions after applying the new technology.

Figure 4B compares the relative changes in climate impact, measured by ATR100, between traditional and advanced low nitrogen oxide and low smoke combustion technologies. The gray cells represent the changes in the new scheme compared with the previous scheme, i.e., the impact of the new technology application on climate. Black whisker lines indicate the degree of uncertainty.

Based on the figures, it can be predicted that D261+ will reduce CO_2_ and water vapor emissions. However, it also increases NO_x_ emissions. D261+ Dropping and D261+ SAF are similar to D261+, but they use DAC, resulting in a significant decrease in CO_2_ emissions for D261+ Dropping, whereas D261+ SAF does not produce any CO_2_ emissions.

In D261 + H_2_, the NO_x_ emissions will be eliminated due to the advanced combustion. Although it also increases the 40% impact of water vapor on climate change, water vapor has a smaller overall impact.

The use of D261+DropIn can help to predict a reduction in CO_2_ emissions. Furthermore, coupling a low NOx and soot combustion chamber with D261+DropIn can significantly lower NO_x_ and soot emissions without affecting CO_2_ emissions. Conversely, using hydrogen in aviation can result in zero CO_2_, NO_x_ and soot emissions but can increase H_2_O emissions. In contrast, choosing D261+SAF, such as e-kerosene, can lead to zero CO_2_ emissions and lower soot emissions. Coupling a low NO_x_ and soot combustion chamber with D261+SAF can strongly decrease NO_x_ and soot emissions, helping to achieve the goal of zero emissions.

## The expected costs of fossil fuels, biomass-derived fuels, and synthetic jet fuels

There has been a lot of discussion about using biomass-derived jet fuels, which are often made from waste materials, for cost-effectiveness. The focus has been on SAFs made from renewable feedstocks, such as kerosene jet fuel. This section compares the expected costs of fossil fuels, biomass-derived fuels, and synthetic jet fuels.

The cost comparison for jet fuel is based on the following:

• DAC-kerosene: PtH_2_ + CO_2_ from DAC
• Bio-kerosene: HEFA, gasification + FT, alcohol-to-jet (AtJ)
• Fossil jet fuel (reference): crude-oil-derived jet fuel, including bandwidth of CO_2_ prices for sensitivity

According to the predictions, the price of domestic DAC-kerosene in the EU-27 region is expected to decline from $132.43/MWhFTL, HHV in 2030 to $77.23/MWhFTL, HHV in 2050. In comparison, the costs in the United States are expected to drop from $121.36/MWhFTL, HHV in 2030 to $71.74/MWhFTL, HHV in 2050. The EU-27 region is likely to benefit from gasoline imports from South America, North Africa, the Middle East, and Australia at local production costs of approximately $123.61–135.75, $88.29, and $70.63/MWhFTL, LHV in the years 2030, 2040, and 2050, respectively.^26^

In the hydropower-assisted scenario, the FTL cost reduction is expected to be $11.04–33.11/MWhFTL, LHV, but it could be greater. However, the cost reduction will decrease over time. The availability of hydropower can help shorten the cost of FTL by approximately 5–10 years compared with the zero-hydropower base case, but the anticipated quantities are relatively low.

It is possible to reduce DAC-kerosene costs by 0%–12% by employing CO_2_ point sources wherever feasible, and the results suggest that availability is also possible. The CO_2_ point sources mainly include biogas upgrading plants, waste incinerators, and cement plants. No CO_2_ transportation costs are calculated if the CO_2_ synthesis plant is assumed to be the CO_2_ point source on site. Ueckerdt et al. estimated levelized costs of e-fuels for 2020–2050; the transportation cost has been included in the case in which hydrogen is produced in a renewable-rich country and shipped ∼4,000 km. Other costs, such as potential taxes and levies or further domestic transport or distribution costs, must be considered.^15^^,^^20^^,^^27^

Bio-kerosene’s cost has been determined using ICCT (2019) data on CAPEX, lifespan, feedstock costs, and additional expenses such as operation and maintenance from IEA (2020). According to IEA (2020), the cost of jet fuel from HEFA ranges from $55.18 to $97.12 per MWh of final fuel ($15.34 and $26.93/GJ, depending on the LHV). European and United States biodiesel will cost around $0.8 per liter in 2022. Jet fuel production from agricultural leftovers using gasification and FT synthesis costs between $35.32 and $87.19 per MWh of final fuel ($9.82 and $24.17/GJ). Jet fuel from lignocellulosic energy crops, such as short-rotation forestry produced by gasification and FT synthesis, costs between $61.81 and $124.70 per MWh of final fuel ($17.21 and $34.65/GJ). The cost is cheap because these pathways are primarily driven by high capital costs and use abundant, relatively cheap feedstocks. No expenses for AtJ are mentioned by IEA (2020). According to IEA (2020), the lower figures are mainly due to the assumption of reduced feedstock prices compared with ICCT (2019).^26^^,^^28^^,^^29^^,^^30^

## Analyzing area demand in the production of various aviation e-fuels

DAC plant cannot be minimal compared with the overall net area demand, and it is negligible compared with the entire gross area demand. The number of facilities required determines the total area needed to produce DAC-kerosene, including the synthesis, DAC, and renewable power plants. The synthesis plants require only a small portion of the overall space, and this approach allows for a comparison of the area demand for the DAC plant with the total area demand. It has been found that the area demand for the DAC plant cannot be minimal compared with the net area demand, but it is negligible when compared with the gross area demand. The area needed for the electricity supply from PV and wind power plants alone reflects this and consists of the following:

• Between 99.2% and 99.8% of total gross area
• Between 86.1% and 99.1% of total net area

It has been discovered that when electricity is generated from onshore wind farms, the highest total gross area demand is observed.^31^

Conversely, the net area demand is lowest when the power is derived from onshore wind farms and highest from photovoltaic (PV) sources.^32^

**Figure 5:**
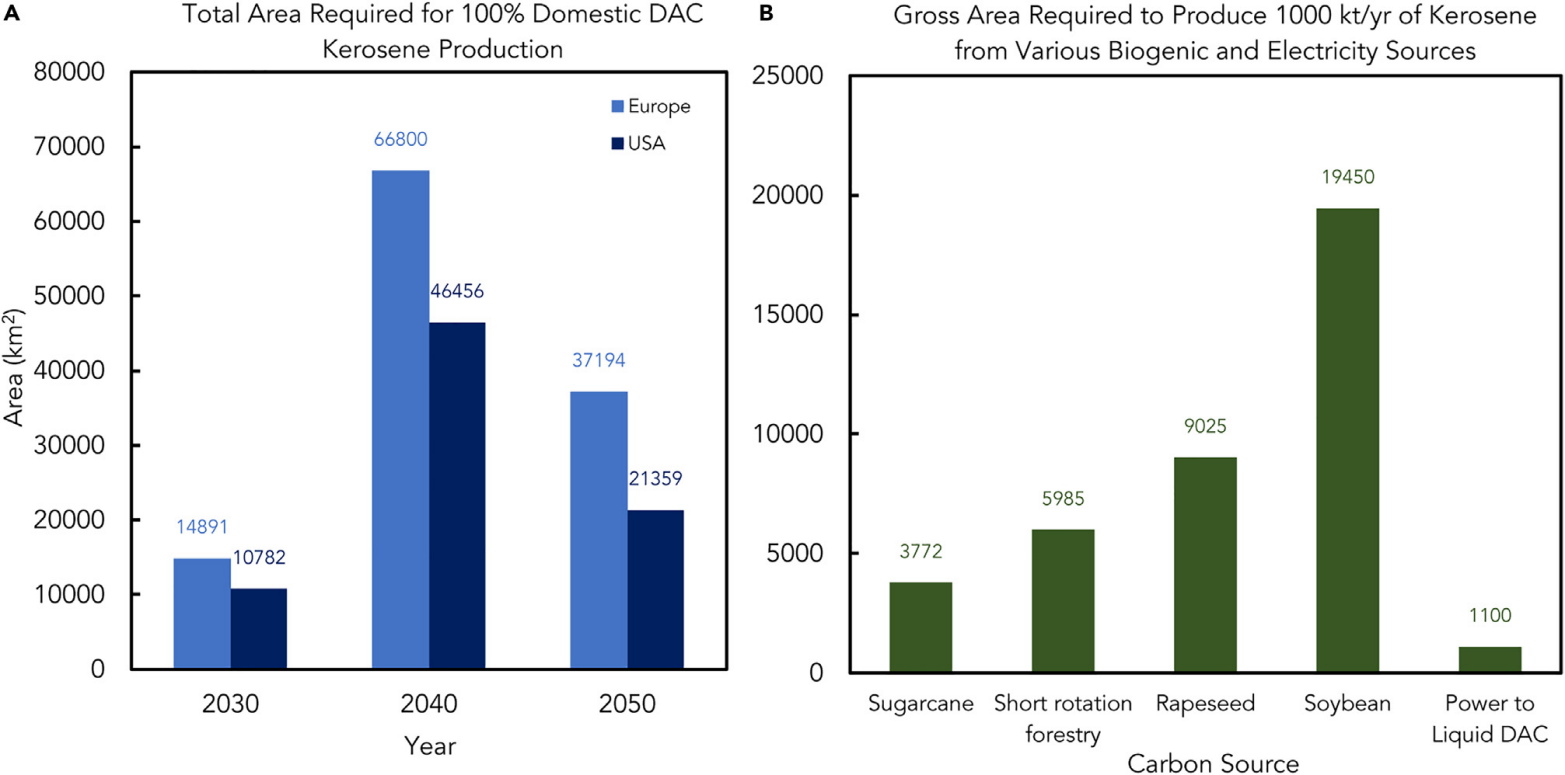
Area demands for production of various e-fuels E-fuel production demands vast renewable energy-rich land. Solar and wind areas are the canvas for production, backed by efficient infrastructure. Advancing technology may transform the process, painting a dynamic future for large-scale production.^33^^,^^34^ (A) Estimated total area required to fulfill the demand for DAC-kerosene in the United States and Europe.^26^ (B) Estimated total area required to produce 1000 kt/y of kerosene from various biogenic and electricity sources.^26^

Moreover, due to the higher capacity factors of on-shore wind and PV single-axis plants in the United States, which are measured as annual averages in likely future installation sites, the area required in the United States is less than that required in the EU-27. Thanks to the improvements in energy conversion efficiency, the area needed for energy generation will decrease from 2030 to 2050 (Figure 5A). The required area peaks in 2040 and is lower in 2050, whereas DAC-kerosene demand is projected to grow continuously until 2040 and decline until 2050. The decline in gross area demand is mainly caused by an increase in the share of electricity from PV (having a lower specific gross area demand than onshore wind power) in the total utility-scale renewable electricity mix and partially by efficiency gains. Figure 5B shows the estimated gross area (km^2^) to produce 1,000 kt/y of kerosene from biogenic and renewable sources. The gross area data are significant when estimating the total area required to meet a specific demand. This helps determine whether sufficient land is accessible within a given region for domestic production. Biogenic sources require more area as there is a direct correlation to the amount of raw material that can be produced to get 1,000 kt/y of kerosene. However, Ptl DAC requires no biogenic sources, so the area required is minimal.

This study determines the amount of land required to produce the volume of DAC-kerosene, assuming it is generated locally without any imports. This is done to calculate the maximum territorial acreage necessary to fulfill the demand for DAC-kerosene in a fully self-sufficient energy system. The renewable power mix composition projection by Bogdanov et al. was published in 2021.

### Comparison between the European Union and the United States

To compare the gross and net areas required for producing 1,000 kilotons of kerosene annually from biogenic and renewable electrical sources in the EU-27 and the United States, technology data for 2030 are used. This comparison considers moderate technological advancements relative to the current state of the art.^35^

The gross area includes the region between solar panels and wind turbines. The area requirements for gross and net energy crops are similar. It is worth noting that the net area for wind power is roughly 1% of the gross area, indicating that 99% of the land between wind turbines is still available for other uses. The gross area information is more relevant for determining if there is enough space in a particular location for domestic production or assessing the overall area needed to meet a certain demand. The net area offers an idea of how fully occupied this area is and how little is left for other activities. Net area needs for DAC-kerosene are negligible compared with bio-kerosene from the feedstocks examined here.^36^

The United States has larger areas with stronger sun irradiation and lower population density than Europe. Consequently, the average number of comparable full-load hours is higher in the United States than Europe. Because energy crops require between 3,772 and 19,450 km^2^ to generate 1,000 kilotons of DAC-kerosene annually, the corresponding gross and net area requirements are lower in the United States, albeit still very low at 11 to 153 km^2^.^37^

## A comparative analysis of production costs between standard and aviation e-fuels

The standard aviation fuel production is large and still growing. In 2019, it had 106 billion gallons for global demand, which will increase to 230 billion gallons in 2050.^38^ This makes the average cost of standard jet fuel stay at a kind of balanced price. For example, the average fuel cost was $3.04 per gallon during March 2022.

Synthetic e-fuel production involves DAC, synthesis, electrolyte technologies, etc. These technologies are not very mature, which causes a higher cost of e-fuels. Based on this, the average cost of e-fuel production is $189 (£44/€50 per liter) per gallon, which is very expensive.^39^

Table 2 compares the cost and production amount between standard fuel and SAF. The following sections will explain the current status of e-fuel production and related pillar technologies.

**Table 2:** A cost and production analysis of standard fuel vs. sustainable aviation fuel

	Average cost (per gallon, 2022)	Average amount of production (per gallon, in US, 2022)
Standard Fuel	$3.14^40^	17,510 million^41^
SAF	$8.67^42^	79.3 million^43^

## The status of current and planned e-fuel production

Nordic Electrofuel, a company based in Norway, is planning a pilot plant to produce 10 million liters of synthetic fuel in 2025 via the FT process at Herøya Industrial Park in Norway with CCU from geothermal.^44^ According to the plant operator, the technology readiness level (TRL) is 8, and the main output fuels are synthetic kerosene and diesel. TRL 8 represents a technology tested and qualified under operational conditions, like large-scale manufacturing. Upon successful implementation, a second production plant is planned for 2030.^45^

Some key players in SAF production include AIR COMPANY, Gevo, Neste. The current annual production of SAF is around 125 million liters, and investment is in place to expand it to 5 billion liters by 2025.^46^ The demand for aviation fuel is projected to be around 35 billion gallons per year, and the goal is to meet 100% aviation fuel demand by 2050.^47^

Table 3 summarizes different companies producing biofuels, showcasing the diversity in production capacity, feedstock, and energy sources used in this industry. For instance, Gevo Inc. has a production capacity of less than 65 million gallons per year, using corn, sugar, and ethanol as feedstock and wind energy for production. In contrast, Johnson Matthey produces 100,000 tons annually using CO_2_ and green hydrogen as feedstock and biomass as an energy source. Table 3 also provides information on the number of plants each company operates and their geographical location. For example, Neste operates five plants across Singapore, Houston, San Francisco, Amsterdam, and Rotterdam, using waste cooking oil and animal fat as feedstock. This table demonstrates the wide-ranging strategies companies employ to produce biofuels, reflecting the breadth and potential of this emerging sector.

**Table 3:** Current status of SAF-producing companies worldwide

Company	Current/Expected production capacity (million gallon/y)	Feedstock	Number of plants	Location	Energy source
Air Company	<1	CO_2_	1	Brooklyn	–
World Energy	250 (Year 2024)	Fats, oil, grease	1	Paramount (California)	–
Gevo Inc	<65	Corn, sugar, ethanol	3 (under construction)	Luverne, Englewood, Northwest Iowa (under construction)	Wind
Neste	33 (Year 2022)	Waste, cooking oil, animal fat	5	Singapore, Houston, San Francisco, Amsterdam, Rotterdam	–
Johnson Matthey	32 (Year 2025)	CO_2_, green hydrogen	1	UK	Biomass
Chevron Lummus Global LLC	36	Yellow grease, brown grease	1	Singapore	Electrolysis
Alder Fuels	2	Sustainable biomass	1	Nevada	Biomass
Sky NRG	30	Biomass	2	Amsterdam, Netherlands	Biomass
Shell Aviation	528 (Year 2025)	Waste oils, agricultural wastes	1	Netherlands	Renewable Resources
Honeywell International	250 (Year 2025)	Cellulosic	1	United States	Biomass
OMV	100 (Year 2030)	Various oils	1	Austria	Biomass, Wind, Solar
ENI	263	Biomass, agricultural waste	1	Italy, Switzerland, France	Biomass, Solar

Adapted from ref.^48^^,^^49^^,^^50^^,^^51^^,^^52^^,^^53^^,^^54^^,^^55^^,^^56^^,^^57^^,^^58^

**Figure 6:**
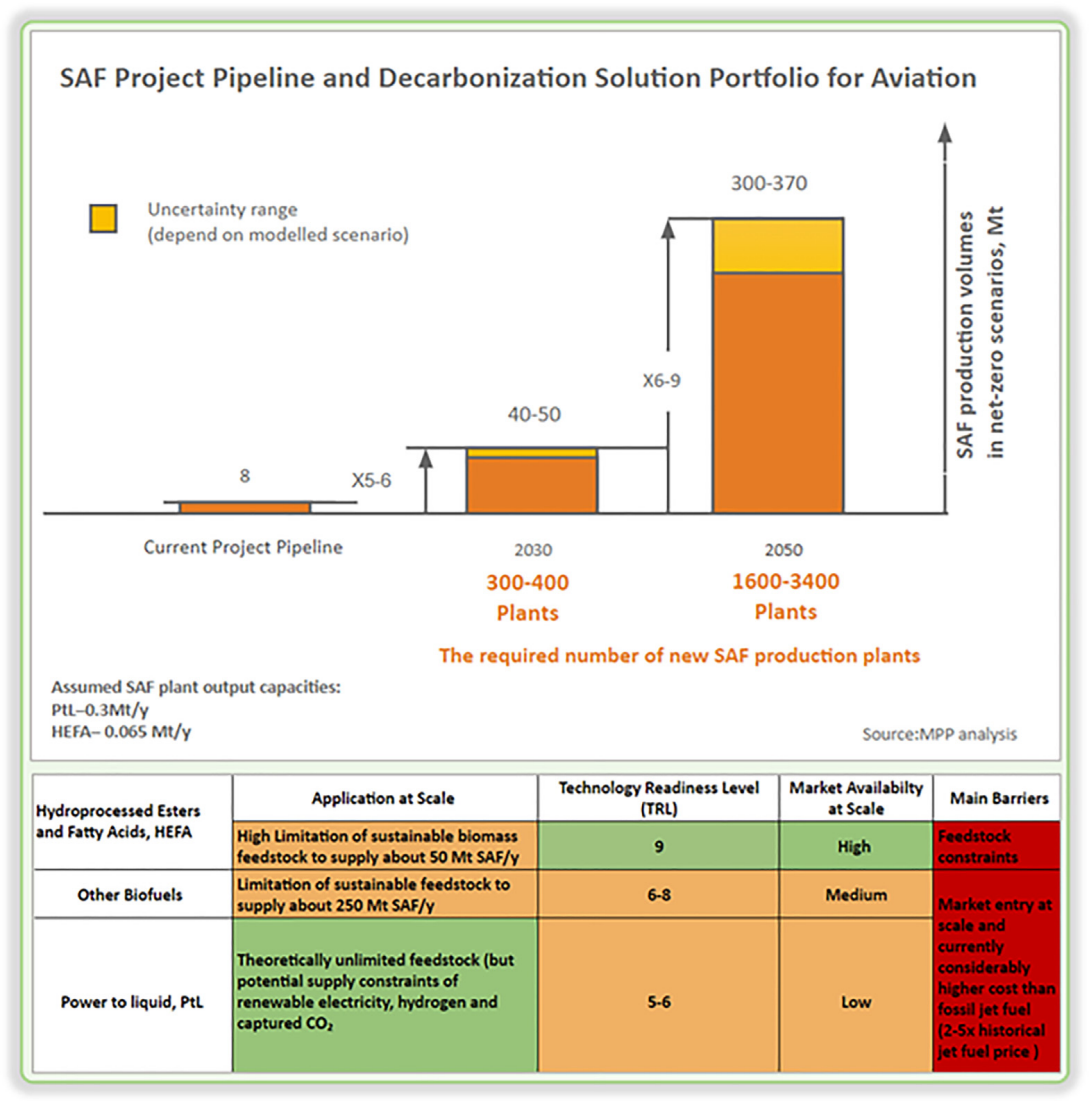
Current and projected SAF portfolio for aviation and their technology readiness levels The SAF project pipeline graph predicts a 5–6x increase in SAF production from 8 to 50 Mt by 2030 to achieve net-zero by 2050. In the Decarbonization Solution Portfolio table, PtL has a theoretically unlimited feedstock supply, but TRL is low compared with HEFA and other Biofuels. This makes this currently expensive to operate at scale. HEFA, hydroprocessed esters and fatty acids; PtL, power to liquid.

To achieve net zero by 2050, SAF and electricity-based (PtL) fuels must be rapidly scaled up, particularly in the 2030s. Aiming for a production target of 40–50 million tons of SAF by 2030 is crucial for this scale-up. This will require investments in around 300–400 new fuel production plants and associated infrastructure within the next two to three years, given the five-year time frame to build and fully operationalize a new SAF plant (Figure 6). Limited sustainable biomass resources necessitate policies prioritizing their use in sectors like aviation, with limited decarbonization alternatives. To expedite the scale-up of bio-jet fuel production, ethanol volumes currently used in the road transport sector could be redirected to aviation as the electrification of cars increases. This can be achieved through the alcohol-to-jet process and by encouraging HEFA (hydroprocessed esters and fatty acids) plants to reduce diesel outputs in favor of jet fuel. Combining these measures could unlock an additional SAF supply of 14–22 million tons by 2030, addressing 25%–50% of the projected SAF demand and setting the stage for the massive scale-up needed in the 2030s to achieve the 2050 net zero target.

### HEFA, other biofuels, and PtL

HEFA has a high application at scale and a technology readiness level (TRL) of 9 (Figure 6), indicating it is a mature technology with significant market availability. However, its main barrier is the limitation of sustainable biomass feedstock, which can only supply about 50 million tons of SAFs annually. Other biofuels, with a TRL between 6 and 8, face medium market availability and are limited by a sustainable feedstock supply of around 250 million tons of SAFs annually. The main challenges for these biofuels are market entry at scale and their considerably higher cost than fossil jet fuel, which is about 2–5 times the historical jet fuel price. PtL fuels, with a TRL between 5 and 6, have low market availability and theoretically unlimited feedstock potential. However, they face potential supply constraints of renewable electricity, hydrogen, and captured CO₂.^45^

## Technological pillars shaping the production of aviation e-fuels

Aviation e-fuel production using DAC and green hydrogen includes two main steps. Firstly, the captured CO_2_ is combined with green hydrogen to produce syngas, a mixture of carbon monoxide (CO) and hydrogen (H_2_). This process is usually achieved through a thermochemical process called the reverse water-gas shift reaction (RWGS). Secondly, the syngas produced in the first step are converted into liquid hydrocarbons using various synthesis pathways, such as Fischer-Tropsch synthesis (FTS) or alcohol-to-jet (AtJ). Catalysts are used in these synthesis processes to convert syngas molecules into longer hydrocarbon chains, which form the building blocks for aviation e-fuels. Hence, technological pillars for aviation e-fuels are DAC, green hydrogen production, and FT e-fuel synthesis using CO_2_ from DAC and green hydrogen from water electrolysis, and they will be discussed in detail in later section.

### Carbon capture technologies

Carbon capture technology has significant potential for mitigating the adverse effects of climate change by decreasing the amount of carbon in the atmosphere and utilizing it to produce synthetic fuels, thereby reducing the dependence on traditional fuels. Currently, there are three primary technologies employed for carbon capture storage and utilization, namely pre-combustion, oxyfuel combustion, and post-combustion technologies.^11^^,^^59^

The process of carbon capture generally involves three main steps: the first step is capturing CO_2_ from the atmosphere, which can be done either by DAC or PSC. The second step involves the transportation of the captured carbon. The CO_2_ is compressed and transported through pipelines, road transport, or ships to a site where it can be utilized or stored. The final step is the utilization or storage of the captured carbon. The compressed CO_2_ can be used as an input to produce carbon, which can then be combined with hydrogen from different processes to make the desired fuel. Alternatively, it can be used in other processes, such as making plastics or building materials like cement. It is also possible to make materials like carbon fiber or graphene. Moreover, the captured carbon can be sequestered and injected deep into rock formations for permanent storage.^60^

The process of carbon capture can be categorized into the following types.

#### Pre-combustion

In the pre-combustion process, conventional fuels like coal or natural gas react with O_2_, with or without steam, to produce synthesis gas, also known as fuel gas or syngas, which is a mixture of carbon monoxide (CO) and hydrogen (H_2_). CO_2_ produced during pre-combustion is available at a higher pressure and can be separated through various physical and chemical absorption processes for storage or utilization. The H2 and CO2 mixture can be separated using physical or chemical absorption techniques with a liquid solvent selective to carbon dioxide. The CO_2_ is then compressed and liquefied at low power requirements for storage or transportation purposes. This also promotes the production of H_2_ as a fuel that can be used in fuel cells (after further purification), transportation, or as a building block in the production of value-added chemicals. The purity of CO_2_ is an important factor to consider. Adsorptive and membrane reactors can increase purity, where the reaction and separation occur in a single unit, reducing energy requirements and by-product formation and increasing overall efficiency. The flexibility of the outputs is an additional benefit where H_2_ production or power generation can easily be switched according to demand. Pre-combustion is theoretically cheaper than post-combustion and oxy-fuel combustion by 38%–45% and 21%–24%, respectively. However, retrofitting current facilities would be required, increasing the cost and complexity, limiting its commercialization.^59^

#### Oxyfuel combustion

Oxyfuel combustion is a process where carbon-based fuel burns in a mixture of recirculated flue gas and pure oxygen (O_2_) instead of air. One advantage of this method is that it makes carbon dioxide (CO_2_) capture and separation easier and has a lower efficiency penalty of only 4%, compared with 8%–12% for the post-combustion route. Another benefit of oxyfuel combustion is that it reduces the amount of flue gases and nitrogen oxide (NO_x_) emissions due to the recirculation of flue gases. The chemical looping method can also increase the net power plant efficiency by 3% with an integrated gasification combined cycle (IGCC). This method can also help reduce capital costs by 10%–18% and electricity costs by 7%–12%.

Oxyfuel combustion can work with both new and old power plants, and it can use any fuel as its feedstock, such as municipal solid waste or lignocellulosic biomass. Combining biofuel with carbon capture and storage (CCS) is known as BioCCS or BECCS. This integration can help in achieving carbon negativity. Studies have shown that in oxyfuel combustion of lignocellulosic biomass, the net electricity production results in emissions of (−0.27 kg CO_2_ MJel^−1^), whereas the integration of carbon capture along with municipal solid waste incineration has led to emissions of (−0.70 kg CO_2_eq kg^−1^) wet waste feedstock.^61^ However, the BECCS approach has some challenges, such as high biomass costs compared with fossil-based fuel, high electricity costs, and lower efficiency. One obstacle in this approach is obtaining pure oxygen, which requires an energy-intensive and expensive separation process in the air separation unit. The addition of air separation, CO_2_ purification, and compression units decreases the cycle efficiency by 9%–13% points due to their energy-intensive nature.^59^ However, the pressurized oxyfuel combustion cycle performs better than the traditional atmospheric cycle and can increase efficiency by 3%.^62^

#### Post-combustion

In this method of capturing carbon dioxide, the CO_2_ is taken from flue gases in a diluted form. The exhaust flue gas emissions must go through denitrification, desulphurization, dust removal, and cooling to prevent solvent degradation before capturing CO_2_ begins. After this, the flue gases are then fed to the absorber that contains the solvent. The scrubbed gas is then washed with water, followed by CO_2_ regeneration.

The captured CO_2_ is then compressed into a supercritical fluid and transported for storage in a geological reservoir or saline aquifers. However, the flow rate of CO_2_ is high, and its concentration is low in flue gas streams, making solvent regeneration energy-intensive. The adsorption route of post-combustion capture is another method, but the only commercially available method is monoethanolamine chemisorption, which is highly expensive in capital and operating costs.

Using membrane separation could be more cost-effective as it requires less energy, has a low carbon footprint, and has low operational costs. It is also easy to retrofit and scale up current plants. However, some disadvantages of using this method include water condensation on the membrane, rapid diminution of selectivity and permeance after operation, and emissions (NO_x_ and SO_x_) that pass through the membrane. Some membranes also suffer from complex temperature adjustments and fluctuations in humidity that cause a drastic change in the transport characteristics of the membrane.

Currently, post-combustion technology is the most widely used carbon capture and storage route. However, due to the dilution of CO_2_ in the flue gas due to the presence of N_2_ in the air, there is a reduction in the partial pressure of CO_2_, which increases the cost of electricity generation by approximately 60%–70% for new infrastructure and by 220%–250% for retrofitting current plants.^59^

#### Point source capture of CO_2_

PSC is a carbon capture technique used at industrial plants, such as chemical production (ammonia, hydrogen, petrochemical), mineral production (cement and lime), natural gas processing, and iron and steel production plants. The process separates carbon dioxide (CO_2_) emissions from the plant’s flue gas or other exhaust stream.

The complexity of capturing CO_2_ from industrial sources varies across sectors, as CO_2_ concentrations vary for each industry. Industrial sources with a highly concentrated stream of CO_2_, such as natural gas processing, fertilizer production, hydrogen production, and ethanol production, require less energy for CO_2_ separation. However, for lower-concentration industrial sources like iron and steel production, cement manufacturing, and petroleum refining facilities, developing carbon capture technologies poses significant challenges due to varying gas compositions, process temperatures and pressures, contaminants, and energy requirements.

#### CO_2_ from ethanol production

In ethanol production, there are two main sources of CO_2_ emissions. During fermentation, carbon dioxide is produced as a byproduct at high purity. CO_2_ is also emitted when fuel is burned, with a relatively low concentration of CO_2_ in the resulting flue gas. Due to the high-purity fermentation stream, ethanol facilities are optimal candidates for CCUS (carbon capture, utilization, and storage). Hybrid capture systems implemented in ethanol plants can capture CO_2_ from both the bioprocessing and heat production streams, resulting in negative emissions.

#### CO_2_ capture from iron and steel production

The primary steel production process involves using iron ore and a technique called blast furnace with basic oxygen furnace (BF-BOF). To reduce greenhouse gas emissions, capture technologies are being developed to capture CO_2_ from the blast furnace gas because it contains the highest concentration of CO_2_ in the steelmaking process. However, the process heaters spread throughout the plant are responsible for a significant amount of CO_2_ emissions, and capturing CO_2_ from them is challenging due to the absence of a common flue stack.

#### CO_2_ capture from cement manufacturing plants

In the manufacturing of cement, the release of CO_2_ occurs mainly during the production of cement clinker, which happens through the process of limestone calcination and fuel combustion for kiln heating. Cement production can combine CO_2_ emissions from processes and combustion in a single stack, making implementing carbon capture, utilization, and storage (CCUS) easier.

#### CO_2_ capture from hydrogen production

Hydrogen production through steam methane reforming (SMR) involves the reaction of natural gas and steam to produce synthesis gas (syngas), a mixture of carbon monoxide and hydrogen gas. A water-gas shift reaction may increase the amount of hydrogen gas in the product gas, which also increases the concentration of CO_2_ in the shifted syngas. During hydrogen production, CO_2_ is emitted from both the process gas and the combustion of natural gas (flue gas). There are three separate sources in an SMR plant from which CO_2_ can be collected or vented: the SMR flue gas, the syngas stream before pressure swing adsorption (PSA), and the PSA tail gas.

In auto-thermal reforming (ATR) plants, hydrogen-rich syngas is produced by partially oxidizing natural gas with either highly pure oxygen from an air separation unit (ASU) or input air. The autothermal (internal) combustion process in ATR plants results in a higher concentration of CO_2_ in the syngas than in the flue gas. Therefore, it is more economical to capture CO_2_ from the syngas stream before it enters the PSA unit in an ATR plant because CO_2_ capture from flue gas is much more expensive and complex due to the low CO_2_ partial pressure and impurities of N_2_/O_2_.^63^

### Direct air capture

The DAC technology is a method used to capture CO_2_ from the air, which results in a concentrated stream of CO_2_. This concentrated stream can be utilized for various applications or stored in geological reservoirs or saline aquifers. The produced CO_2_ is versatile and can be used in multiple ways. For instance, it can produce E-Kerosene, a more sustainable fuel source. The two most extensively developed processes are the liquid solvent and solid sorbent DAC. These processes have been described in the following sections.

#### Solid DAC

Solid-sorbent-based chemically functionalized DAC with amines is one approach to DAC. Solid sorbent options such as materials with affinities for CO_2_ and materials with surface functionalization purposed for CO_2_ capture are used. Researchers are looking for materials that have a good affinity for CO_2_ and are cost-effective. MOFs, activated carbon, silica gels, cellulose, zeolites, and carbon nanotubes are materials under research for this purpose.^12^^,^^13^^,^^64^

**Figure 7:**
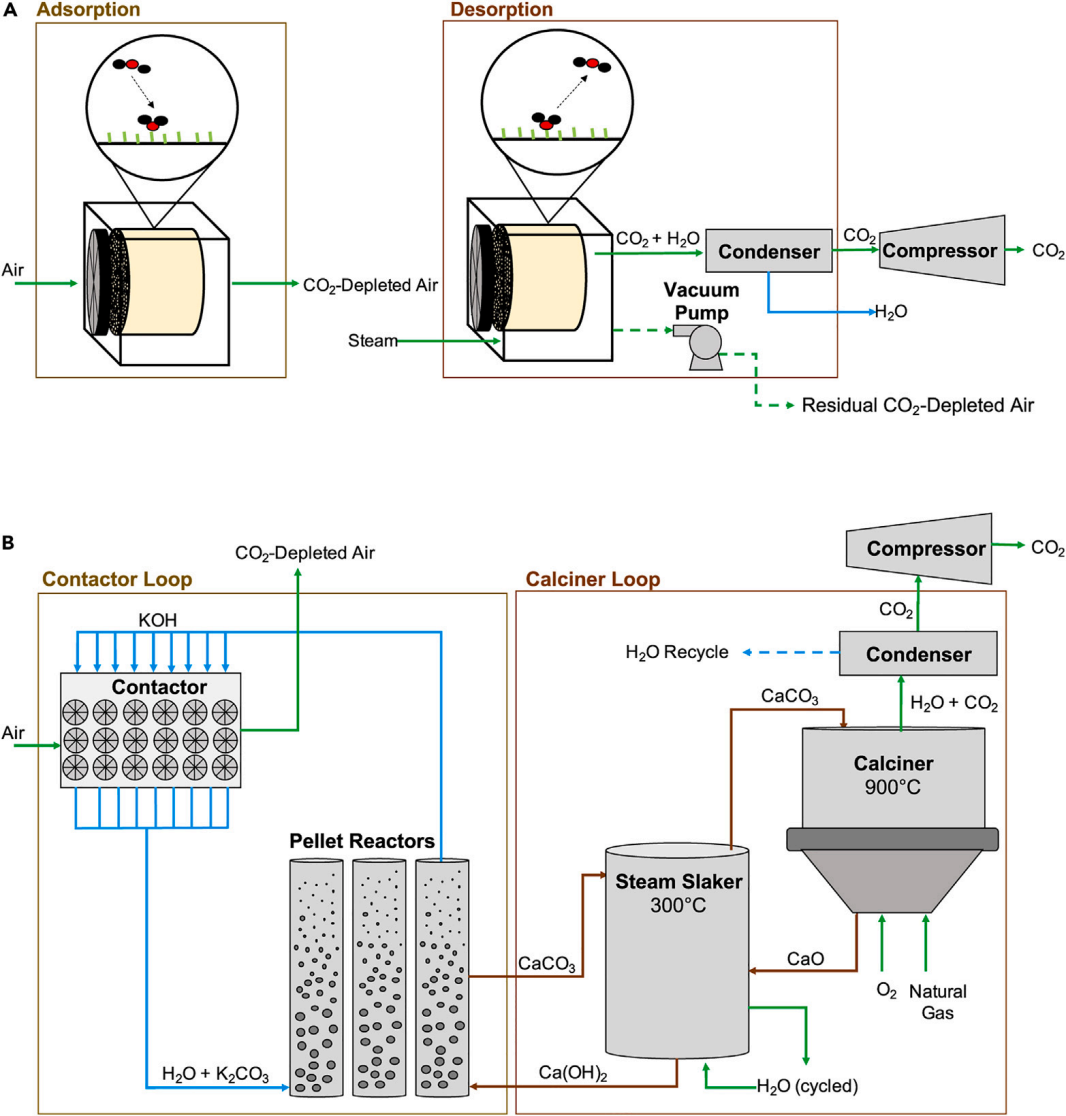
The process flow diagram for solid and liquid DAC The process flow diagram for solid and liquid DAC to capture carbon emissions effectively utilizes sorbents or solvents to selectively absorb CO_2_. The absorbed gas is then purified and concentrated through desorption or regeneration processes. These methods are essential for mitigating carbon emissions and promoting sustainable solutions in the battle against climate change. (A) Representative process flow diagram for solid sorbent DAC.^65^ (B) Representative process flow diagram for the solvent process.^65^

Figure 7A represents a process flow diagram of the stationary bed solid sorbent DAC process. In this process, air is pushed through the contactor unit using fans, and the CO_2_ adsorbed onto the solid sorbent at ambient conditions. Once the solid sorbent is saturated with CO_2_ or has reached the desired CO_2_ uptake, the CO_2_ is collected by desorbing the solid sorbent. At this stage, the contactor is closed off from the surrounding environment. A vacuum pump removes residual air from the contactor to prevent dilution of the produced CO_2_ by residual oxygen and nitrogen in the contactor and minimize amine degradation from the air. A vacuum pressure of approximately 30 mbar is usually applied. The vacuum stage reduces the temperature requirements for regeneration. The steam is then sent into the contactor to heat the material to the regeneration temperature (roughly 80°C–120°C). The steam also flushes the released CO_2_ from the contactors, which is then separated from water in the condenser and sent for compression. Finally, the CO_2_ is transported, stored, or utilized for various processes.^65^

#### Liquid DAC

In the solvent DAC method, KOH is mixed in an aqueous solution, dissociating into ionic K+ and OH−. The process involves a dilute stream of CO_2_ being brought into contact with the aqueous phase, which produces K_2_CO_3_. The stoichiometry of K_2_CO_3_ indicates that two KOH molecules are needed to separate one CO_2_ molecule.

The liquid solvent DAC system has two loops: the contactor loop and the calciner loop, as shown in Figure 7B. In the contactor loop, air is forced horizontally through long contactor units (roughly 200 m by 20 m by 5–8 m in dimension) to capture about 1 Mt of CO_2_ per year. In recent designs of the L-DAC system, the air is pulled horizontally through the packing material using a fan, and the air exits vertically. In the contactors, a 2 M KOH solution flows vertically through the packing material, which reacts with the CO_2_ in the air to form potassium carbonates in solution (K_2_CO_3_). After this stage, the solution is pumped to a central regeneration facility, where the K2CO_3_ undergoes an anionic exchange with calcium hydroxide [Ca(OH)_2_] in the pellet reactors to form calcium carbonate (CaCO_3_) and regenerate the KOH solution. The regenerated KOH solution is then pumped back to the contactors for use in another cycle.

The pellet reactors produce larger CaCO_3_ crystals simultaneously through controlled precipitation reactions to produce CaCO_3_ pellets larger than 0.85 mm. The produced CaCO_3_ is then fed into a steam slaking unit, where heat from the calciner products is used to dry the CaCO_3_ from the pellet reactors before they are fed into the calciner. In the calciner, the CaCO_3_ is heated to 900°C, where it undergoes a decomposition reaction to form calcium oxide (CaO), water, and CO_2_.

The calciner is internally fed with natural gas and oxygen to obtain the required temperature, which results in a gaseous mixture consisting primarily of CO_2_ and water. The CaO is then fed into the slaking unit, which is hydrated to Ca(OH)_2_. From here, the Ca(OH)_2_ can be fed back into the pellet reactors for the anionic exchange. The gas produced at the calciner is sent through a condenser to remove most of the water, and the resulting CO_2_ is compressed.

The energy requirement for this process to capture one ton of CO2 is equivalent to 8.81 GJ natural gas or 5.25 GJ natural gas coupled with 366 kWh of electricity. Carbon Engineering estimates the capture cost for this process to be between $94 and $232 per ton of CO_2_ captured. Replacing fossil fuels used for heat production with other sources can further reduce carbon emissions and may also lower the cost of the process, which is a major hindrance to using carbon capture processes today.^65^

#### DAC’s status

In recent years, there has been a lot of focus on reducing carbon in the atmosphere and finding alternative fuel sources. As a result, carbon capture technology has made significant progress. However, the widespread application of this technology is still hindered by the high capital investment required and the enormous infrastructure and energy demands.^10^^,^^11^^,^^65^^,^^66^ Currently, 35 commercial capture facilities operate globally, with a total annual capture capacity of nearly 45 Mt CO_2_. Several new carbon capture facilities have started operating in recent years, such as the Gorgon CO_2_ injection project in Australia (2019), two capture facilities linked to the Alberta Carbon Trunk Line in Canada (2020), the first large-scale bioenergy with carbon capture project (BECCS process) in Japan (2020), and two capture facilities in China at the Sinopec Chemical plant and the Guohua Jinjie coal power plant (2021). In 2021, a positive final investment decision (FID) was made on six CCUS projects that aim to capture about 6.5 Mt CO_2_ per year once operational. Developers of these projects estimate that more than 200 new capture facilities could be operating by 2030, which will capture over 220 Mt CO_2_ per year.^66^

**Figure 8:**
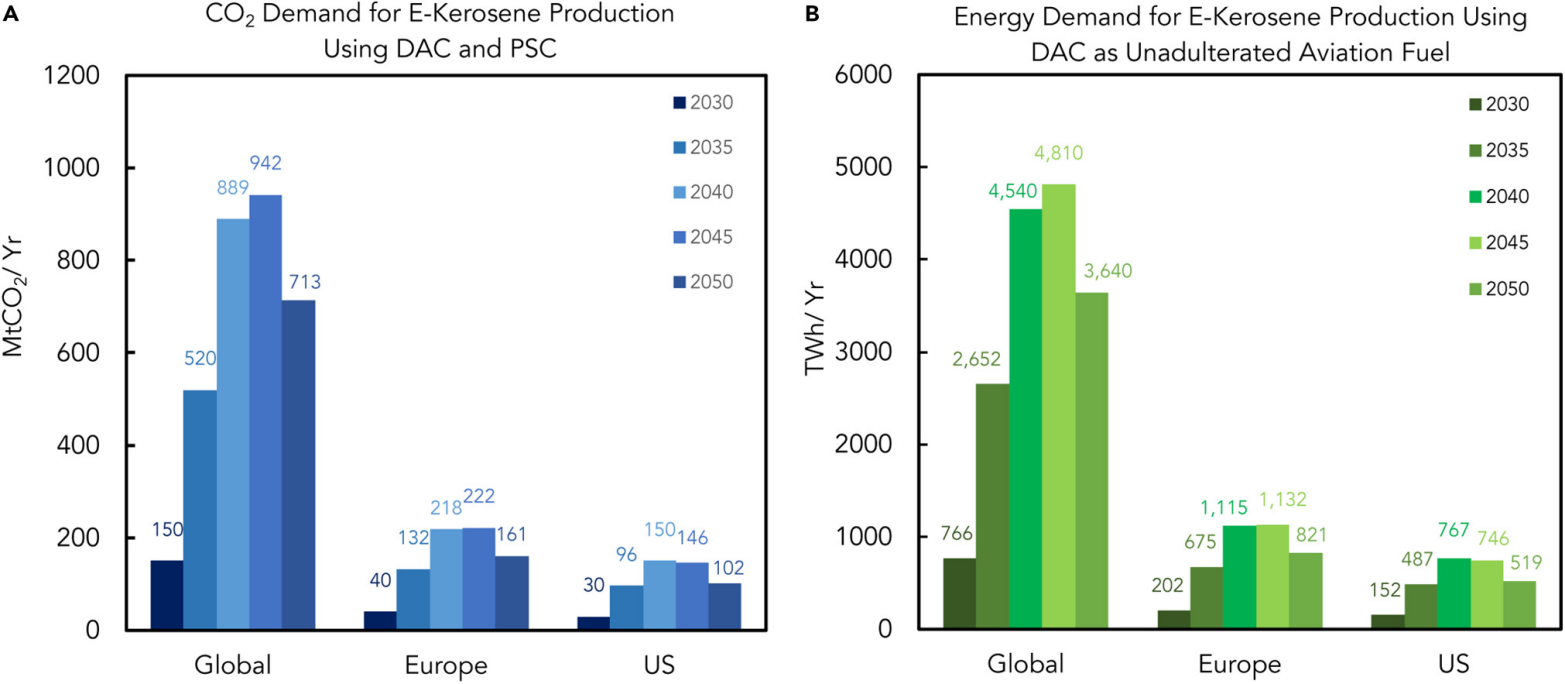
Projected CO_2_ demand for e-kerosene production and related energy demand in the operation of DAC (A) CO_2_ demand for e-kerosene production using DAC and PSC (in MtCO_2_/y). E-kerosene production via DAC and PSC reduces emissions, repurposes atmospheric CO_2_ as a feedstock, and showcases innovative strides in cleaner energy solutions.^26^ (B) Energy demand for e-kerosene production using DAC for aviation fuel production (in TWh/y). E-kerosene for aviation fuel is produced via direct air capture (DAC) by capturing CO2 from the air and synthesizing it into kerosene. This process demands significant energy, with DAC requiring considerable input for carbon capture. Achieving a sustainable alternative to traditional aviation fuel requires balancing this energy equation, emphasizing the delicate dance between innovation and efficiency in pursuing greener skies.^26^^,^^35^^,^^67^

Figure 8A describes the CO_2_ demand for the production of e-kerosene using DAC and PSC. This graph suggests that 942 Mt of CO2 per year would be required by 2045 to produce the necessary amount of e-kerosene to sustainably satisfy the fuel needs of the entire aviation industry. This suggests that a major ramp-up is required, meaning that new carbon capture plants would need to be set up, which would require high capital investment for research on different aspects of the carbon capture process and expenditure for setting up the plants and maintaining it.

There are currently many projects under development worldwide aimed at capturing CO_2_ emissions.^68^ Over 30 countries are participating in these projects, with over 10 in Indonesia, Malaysia, and Thailand alone. By 2030, these countries could have a total capture capacity of around 15 million metric tons of CO_2_ annually.

In China, the Sinopec Qilu Petrochemical project was completed in January 2022, and the first Chinese offshore CCUS project was completed in June 2022.

In the United States, there are roughly 80 projects currently underway that are expected to be operational before 2030. The country’s CO_2_ capture capacity could increase by a factor of five from over 20 million metric tons of CO_2_ to over 100 million metric tons of CO_2_ per year.

Canada currently has around 15 projects in various stages of development.

In Europe, around 50 projects are being developed to connect industrial clusters to CO_2_ storage hubs. These projects could capture nearly 70 million metric tons of CO_2_ annually by 2030 in countries such as Norway, the United Kingdom, the Netherlands, Sweden, and Denmark.

In the Middle East, at least four projects are in development in addition to the four already operating. Bahrain has announced plans to deploy CCUS at an aluminum plant, making it the first application of CCUS to aluminum. In Qatar, construction continues on the North Field East LNG project, which will expand Qatar’s CCUS capacity from over two million metric tons of CO_2_ per year to five million metric tons of CO_2_ by 2050.^65^

Several promising technological innovations aim to reduce the cost of CCUS for power generation. These innovations include the following:

• The Drax bioenergy CO_2_ capture pilot project: this groundbreaking initiative is the first to capture CO2 from a power plant that runs on 100% biomass feedstock. There are plans to scale up to a commercial-size project by 2027.
• NET Power’s 50 MW clean energy plant: this natural gas-fired power plant is unique in its Allam cycle technology, which employs CO_2_ as a working fluid in an oxyfuel supercritical CO_2_ power cycle. This innovative approach is expected to reduce costs associated with carbon capture, which has been a major obstacle to the commercialization of CCUS^67^

The aviation industry is expected to be one of the largest beneficiaries of DAC of CO_2_, where sustainably produced fuel such as e-kerosene will replace currently used fossil fuels. As the sector increasingly relies on PtL technology, there will be a greater need for carbon capture technologies. By 2050, the carbon capture capacity must be scaled up to 490–730 Mt/y. This can be achieved through DAC or pressure swing adsorption (PSC). PSC will be needed to start the PtL production before DAC becomes available for large-scale production. Capturing CO_2_ from air is projected to be about three times more expensive in the long run, with $100–$300 per ton of DAC-CO_2_ compared with $50–$100 per ton of PSC-CO_2_.

Decarbonizing the industrial sector will require assessing the technology readiness level (TRL) of various CCUS techniques. Pre-combustion (natural gas processing) is the only capture technology that has reached commercial scale (TRL-9). Other capture technologies such as adsorption post-combustion, oxyfuel combustion (coal power plants), pre-combustion (IGCC), membrane polymeric (natural gas), BECCS technology, and DAC are in the demonstration scale (TRL-7). Techniques such as membrane polymeric (power plants), post-combustion (biphasic solvents), chemical looping combustion, and calcium carbonate looping technologies are in pilot-scale (TRL-6) and the transport technology for the captured carbon, onshore and offshore, on a commercial scale (TRL-9).^59^

Several companies are actively involved in CCUS research and development, which significantly impacts reducing carbon emissions. Following are some of the companies leading the way in this area^69^:

• AirCapture: the company provides modular and on-site systems that supply CO_2_ directly to their customers' production lines. Currently, they are targeting the food and beverage industry, with plans to expand into carbon value markets. These markets include chemicals, plastics, rubber, carbon fiber, and synthetic fuels. They have collaborated with a fertilizer company to transform the captured CO_2_ into value-added chemicals and a cement manufacturing plant to reduce carbon emissions by 2,000–3,000 tons and eliminate 500 tons of atmospheric CO_2_ yearly per production line.
• Capture6: the company has developed technology for DAC, using sources like seawater and focusing on water treatment sites. They use the existing infrastructure at desalination plants and water recovery systems to simplify the DAC process and convert carbon dioxide into carbonates. Additionally, the company utilizes excess salty water, which would otherwise be returned to the ocean, to create a CO2-removing solvent. This results in the recovery of up to 70% of the water treatment rejected, creating value for both carbon removal and water availability.
• CarbonCapture Inc.: this is an innovative company that uses molecular sieves, low-cost renewable energy, and purpose-built systems to capture carbon dioxide. The company is currently working on a project called Bison in Wyoming, which is set to become the largest DAC plant in the world. The goal is to capture and store five million tons of CO_2_ annually by 2030. The plant is expected to become operational in 2023, with a removal capacity of 12,000 tons, which will be increased to 200,000 by 2026. The S-DAC setup uses renewable energy and performs a cyclic process that filters carbon dioxide from the atmosphere and injects it into nearby wells. Due to geography, Wyoming is ideal for injecting the captured carbon into wells.
• Carbon Engineering: the company develops DAC and storage facilities along with Air to Fuel technology, which involves removing CO_2_ from the air and creating synthetic fuel. They use L-DAC systems for carbon capture and storage. In the Air to Fuel process, the CO_2_ produces synthetic crude that can be processed into gasoline, diesel, and jet fuel. This technology can work in existing vehicles and transportation infrastructure without any modifications. Water is split into hydrogen and combined with captured atmospheric CO2 to make electrolysis fuels. Their first pilot was launched in 2015 in Squamish, British Columbia, Canada, where the facility captures approximately one ton of atmospheric CO_2_ daily. The company has partnered with Occidental Petroleum (Oxy) and 1PointFive to construct a massive DAC plant in the Texas Permian Basin capable of initially capturing 500,000 tons of CO2 per year, which can be scaled up to 1 million tons of CO_2_ annually.
• Climeworks: small collectors extract CO_2_ from the atmosphere and can be configured for any plant size. Air is drawn in using a fan located inside the collector. The air then passes through a filter inside the collector, which traps carbon dioxide particles. Once the filter is whole of CO_2_, the collector closes, and the temperature rises to approximately 100°C. This causes the filter to release the CO_2_ that has been collected. The captured carbon is stored permanently in the ground by converting it into stone. In May 2017, Climeworks launched the world’s first commercial project to filter CO_2_ from the ambient air in Hinwil. In October of the same year, they started a project at the Hellisheiði Power Station in Iceland. In September 2021, Climeworks’s Orca carbon capture plant began operations. This facility is the largest DAC plant in the world and captures 4,000 metric tons of CO_2_ per year. Climeworks has announced plans for a larger plant called “Mammoth,” which will capture 36,000 million tons of CO_2_ annually.
• CO_2_Rail: CO_2_Rail is a US startup that uses existing railway infrastructure to capture carbon dioxide from the atmosphere. They achieve this by equipping trains with DAC units. The company’s innovative carbon removal solution revolves around its regenerative braking system, which converts excess heat energy from the train’s operations into electricity. The generated power is stored in a 2,400 kWh battery under the rail car. CO_2_Rail utilizes a revolutionary concept, taking advantage of the train’s natural speed to completely circumvent the need for fans. Due to the train’s movement, air can move at a rate of 15,000 cubic meters per minute. The captured carbon is then converted into a liquid using liquid sorbents and stored in a 15-ton cryogenic storage tank. Thanks to the simplicity of the system and the low initial investment costs of the technology, the cost of capturing CO_2_ is as low as $50 per ton.
• Mission Zero Technologies: the company uses an ion-selective electrochemical separation process to efficiently capture CO2 from the atmosphere and continuously concentrate it as a pure gas. This process consumes 3–4 times less energy than existing thermal regeneration approaches. The company has leveraged mature technologies such as cooling towers and electrochemical water purification to develop this process. In 2022, the company partnered with a carbon mineralization company called 44.01 to collaborate on Project Hajar, located in the Al Hajar mountains of Oman. The captured CO_2_ is dissolved in water and sequestered underground peridotite rock formations.
• Sustaera: a startup in the United States is using easily accessible natural minerals for its CO_2_ sorbents, making use of existing supply chains and manufacturing infrastructure. Their innovative design is claimed to be suitable for any location worldwide and performs well in various levels of humidity and temperature. They achieve this while using less than 100 acres of land to sequester a million tons of CO_2_.
• Valiidun: Valiidun was established in 2022 and utilizes proven DAC technology to cut the costs per ton of decarbonization by half. It mainly focuses on constructing large-scale DAC sites to reduce operating expenditures through economies of scale. Valiidun is currently in the process of securing a grant to build a DAC facility in Appalachia, a region that is striving to revive its economy after a long period of dependence on coal mining.
• Verdox: a new electric system has been designed to improve the efficiency of removing CO_2_ from gases. Unlike traditional methods that use heat to eliminate CO_2_, this system uses a specific voltage to release the captured CO_2_ from the material. The result is a more energy-efficient process that does not require heat or water. In addition, the company has developed a new type of plastic that can selectively trap carbon dioxide from a mix of gases, including exhaust fumes. This material uses electricity to capture CO_2_, making it far more energy-efficient than existing alternatives. Operating requires at least 70% less energy, making it a significant advancement in the field.

Significant progress has been made in developing efficient and cost-effective carbon capture technologies. These technologies employ various methods to capture, store, or use carbon for other purposes. However, increasing the number of DAC plants is necessary to meet future demands. Achieving this will require substantial investments, and many technology entrepreneurs are investing heavily in CCUS technologies. These developments will ultimately contribute to the goal of reducing carbon emissions and promoting the use of sustainable fuel alternatives.

## Energy demand for e-kerosene production using DAC

Carbon capture is a process that requires a lot of energy. Two approaches, solid sorbent (S-DAC) and liquid solvent (L-DAC), use about 80% thermal energy and 20% electricity. Thermal energy is needed to regenerate the sorbent and release CO_2_, making up most energy requirements.

For S-DAC, electricity is needed for the contactor fans to overcome the system pressure drop and the vacuum pumps to remove residual air during regeneration. For L-DAC, electricity is required for contactor fans to overcome the system pressure drop, pellet reactors, steam slackers, and filtration units. Both processes must have a predetermined pressure drop value and a specific amount of CO2 to be removed to determine energy requirements.^65^

It has been observed that the DAC sorbent process requires around 6 GJ of thermal energy per ton of CO_2_ and approximately 1.5 GJ of electricity per ton of CO_2_.^70^ However, some sorbents have lower regeneration energy requirements, which can reduce the thermal energy requirements to about 3 GJ per ton of CO_2_ and even as low as 1 GJ per ton of CO_2_.^71^ On the other hand, the solvent process has thermal energy requirements ranging from 5.25 to 8.1 GJ per ton of CO_2_, which mainly depends on how much heat integration is achieved from the calcination process. Additionally, electricity demands range from 1.3 to 1.8 GJ per ton of CO_2_, depending on the variations in packing material and contactor configuration used.^72^

It can be concluded that the energy requirements for the S-DAC and L-DAC processes are similar. However, the temperature needed for these processes differs significantly. The solid sorbent system requires a temperature range of 80°C–130°C. This temperature range can be achieved through industrial waste heat or other low-quality thermal energy sources. Heat pumps can also attain these temperatures but require more electricity than other methods, increasing electricity consumption beyond 20%. Despite this, the high coefficient of performance of heat pumps would still result in lower electricity consumption compared with resistive heating.^66^

Decomposing CaCO_3_ into CaO and CO_2_ in a liquid solvent system requires heat at around 900°C. Therefore, solid and liquid systems should be paired with different thermal energy sources for optimal efficiency. In addition to efficiency, the cost of energy resources and greenhouse gas emissions also play an important role.^11^^,^^66^

Similar to the DAC process, refineries in the United States require a significant amount of thermal energy and electricity. The refineries consume approximately 3,000,000 billion BTU of energy annually, equivalent to about 880 TWh annually. However, if the same amount of energy was diverted toward a DAC facility that requires around 300 MW of consistent energy to capture 1 MtCO_2_ per year, the same energy could capture 370 MtCO_2_ per year. Therefore, scaling up DAC and increasing carbon capture is a feasible solution. Reducing the energy consumption for DAC through new materials, better techniques, and designs can further increase the amount of carbon capture achievable with the same amount of energy.^65^

The graph in Figure 8B illustrates the energy required to produce e-Kerosene, assuming that all aviation fuel requirements are met with e-kerosene for every year under consideration. As per the graphs, the worldwide energy demand for e-kerosene will peak in 2045 at 4810 TWh per year.^26^

This is a significant amount of energy, and if produced using fossil fuels, it will result in many carbon emissions being released into the atmosphere. This would ultimately defeat the objective of implementing CCUS. Therefore, it is essential to meet this energy demand using alternative renewable energy sources such as wind and solar energy to avoid the adverse effects of carbon emissions.

### Modularity

Modularity refers to the ability of a system to be divided into smaller units. The level of modularity in an engineered system, like DAC, can vary. High modularity makes it easier to mass-produce the system and quickly implement design or manufacturing improvements. Modularity can also help identify the minimum feasible scale a plant can operate.

Compared with the L-DAC route, S-DAC displays higher modularity. Climeworks' solid-sorbent-based process uses segmented contactor configurations to enable the regeneration of individual units. A single contactor unit can capture approximately 50 tCO_2_ per year, and more contactor units can be added to scale up the system. Global Thermostat’s configurations include containerized modules operating within the 1,000–4,000 tCO2 per year range, with the first pilot plant having a capacity of 1000 tCO2 per year.^11^

The partition-based modularity of S-DAC is not seen in the L-DAC system due to its highly integrated nature. In L-DAC, contactors feed CO2-saturated solution to a central regeneration unit, making the minimum feasible scale of the system much larger than that of S-DAC. However, the pellet reactor and air contactor used in L-DAC can be scaled down to achieve capacities of around 10 ktCO_2_ per year with consistent capital costs of up to 100 ktCO_2_ per year.^71^ For large-scale operations, L-DAC is much more economical above the 100 ktCO_2_ per year value because the larger process equipment, such as the calciner and slacker, reaches an economic optimum at approximately 1 MtCO_2_ per year.^73^ Modularity is an essential aspect of the L-DAC system, segregating the operations by function and helping make the process more efficient.^65^

It is crucial to meet the energy demand required to produce sustainable fuel alternatives using renewable energy sources to ensure that the net carbon emissions become negative. Employing efficient techniques and suitable equipment to generate energy that meets the temperature requirements of L-DAC and S-DAC will significantly reduce losses and simplify the process, leading to reduced costs.

### Fischer-Tropsch technology

FT technology is a process that converts carbon sources (like natural gas, coal, or biomass) into hydrocarbons that can be further processed into diesel and jet fuel. FT technology has been utilized in various applications for decades, including producing aviation fuel. Its effectiveness in this regard is widely recognized. However, FT plants are typically large-scale and capital-intensive, requiring significant investments and long lead times for construction. Although the efficiency of FT technology has been improving steadily over the years, the challenge remains to find low-cost and sustainable carbon sources and to deploy carbon capture and storage (CCS) technologies to mitigate the greenhouse gas emissions associated with the production process. Sasol, Shell, ExxonMobil, and BP are some of the key players in the FT technology market (Table 4). The capacity of FT plants can vary significantly based on their geographical location, the type of feedstock used, and the technology utilized. For instance, the Sasol Synfuels plant in South Africa, which uses coal as its feedstock, can produce up to 160,000 barrels per day (bpd), whereas the Pearl GTL plant in Qatar, which employs natural gas as its feedstock, can generate up to 140,000 bpd. Meanwhile, other FT plants can have capacities ranging from a few hundred bpd to tens of thousands of bpd.

• Sasol: the South African company Sasol has been a major player in the FT industry for many years, with several large-scale FT plants in operation^74^
• Shell: Shell operates the Pearl GTL plan, one of the world’s largest FT plants^75^
• ExxonMobil: ExxonMobil has been involved in FT research and development for many years and operates several pilot-scale plants.
• Synfuels China: Synfuels China operates several FT plants in China, including a 100,000-bpd plant in Ningxia.
• Chevron: Chevron has been involved in FT research and development for many years and operates several pilot-scale plants.^76^^,^^77^

**Table 4:** Current status of Fischer-Tropsch Industries worldwide

Key players	Location	FT plant	Number of plants	Feedstock	Capacity (million barrels per day)
Sasol	South Africa	Sasol Synfuels	2	Coal	0.16
Shell	Netherlands	Pearl GTL (Gas-to-Liquids)	1	Natural Gas	0.14
Exxon Mobil	United States	Pearl GTL	1	Crude Oil, Natural Gas Liquids (NGLs)	4.4
INEOS (INspec Ethylene Oxide Specialties)	Switzerland	INEOS Oxide Oligomers	1	Natural Gas	0.55 Mt/y
Qatar	Qatar	Oryx GTL	1	Natural Gas	0.034
Nigeria	Nigeria	Escravos GTL	1	Natural Gas	0.033
South Africa	South Africa	Mossel Bay GTL	2	Natural Gas	0.022

#### Production status

FT technology is currently used commercially in several countries, including South Africa, Qatar, China, Malaysia, and Indonesia. According to a report by Technavio, the global FT technology market was worth $3.7 billion in 2020 and is projected to grow at a compound annual growth rate (CAGR) of 7.54% from 2021 to 2025. The market is expanding due to the rising demand for synthetic fuels, abundant natural gas resources, and the need to reduce carbon emissions.

**Figure 9:**
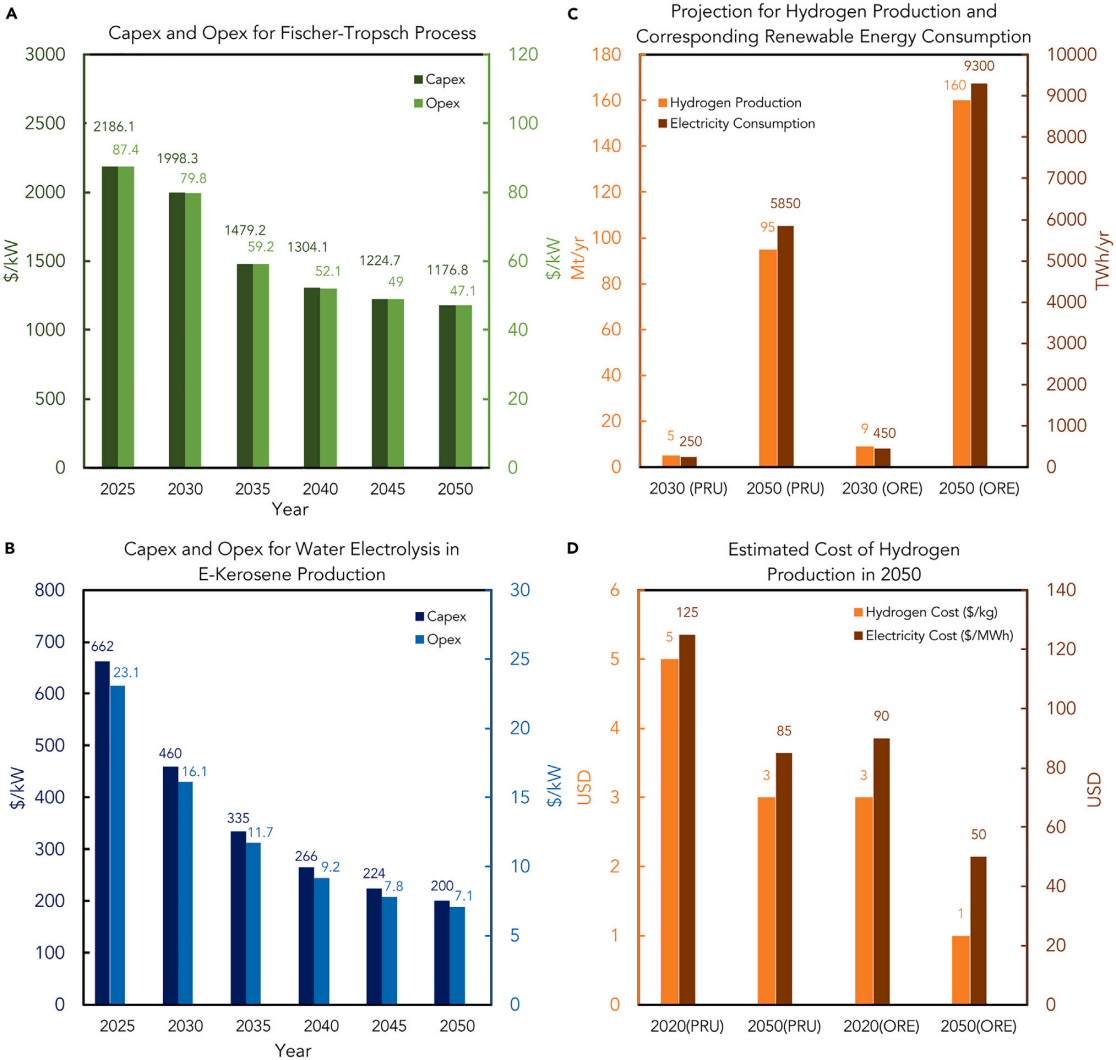
Projected cost for FT process and hydrogen production (A) Projected Capex and Opex for Fischer-Tropsch plants until 2050. (B) Estimation of cost of hydrogen production in 2050 (left frame: PRU and right Frame: ORE), sourced from (MAKING NET-ZERO AVIATION POSSIBLE, 2022).^45^ (C) Projection of green hydrogen production and the corresponding renewable electricity consumption in 2030 and 2050, sourced from (MAKING NET-ZERO AVIATION POSSIBLE, 2022).^45^ (D) The financial assumption of water electrolysis in e-kerosene conversion, which is the capital cost of establishing and operating the plants to produce green hydrogen in the future.^35^

Figure 9A displays the expected values of various parameters for the FT Technology from 2025 to 2050. The parameters include capital expenditure (Capex) and fixed operating expenses (Opex fix), and their respective units are provided for each year.

Figure 9A depicts the capital expenditure and fixed operational costs in dollars per kilowatt of the FT process in five-year intervals from 2025 to 2050. These figures are calculated assuming constant operational variable cost, heat production, electricity, carbon dioxide, and hydrogen consumption as described in ref.^26^^,^^35^

Estimates show that by 2050, both the capital and operational variables' costs will decline by 46.2%, with most of the drop occurring by 2035 at 32.3%.^78^

“Capex” refers to the capital expenditure cost, the initial cost of building the facilities used for water electrolysis (Figure 9B). On the other hand, “Opex” represents the fixed operating cost when running these facilities. The data illustrate the cost in USD per kilowatt in five-year intervals from 2025 to 2050. The cost of both Capex and Opex decreases every five years. By 2050, the capital expenditure cost is expected to decrease by almost 70% compared with 2025, whereas the fixed operating cost would also decrease significantly. The most significant drop in Capex (30.5%) and Opex (30.3%) is expected to occur during the interval from 2025 to 2030, during the development of e-kerosene conversion by the middle of this century. Therefore, significant technical or financial breakthroughs can be expected in the next decade.

### Green hydrogen production

Green hydrogen is widely used to produce sustainable e-kerosene for the aviation industry. It can even be used directly for electricity generation in aero engines. However, over 90% of traditional hydrogen production relies on fossil fuels like natural gas. On the other hand, green hydrogen is produced by splitting water (H_2_O) via an electrolytic process using renewable electricity. As the use of e-kerosene in the aviation market continues to expand, clean electrical power is predicted to become the primary source of energy for producing e-fuels, including hydrogen itself.^79^

Currently, green hydrogen has little impact on the global market. However, its supply must increase 6-fold to achieve net-zero emissions in the energy sector by the middle of this century, according to the IEA 2021a report. The production process of green hydrogen is very energy-intensive, making it an expensive option. Furthermore, its production is also limited by the amount of emission savings sourced from the input electricity. On average, input electricity accounts for up to 90% of hydrogen costs, depending on technology. Therefore, the availability of cheap renewable electricity will be a crucial factor in green hydrogen’s large-scale and low-cost production. It is worth noting that the declining costs of solar PV and electrolyzer capex are leading to an increasing worldwide dominance of PV in supplying electricity for green hydrogen production. This trend is expected to continue and even intensify by 2050, as per the Kopf & Craglia report.^80^^,^^81^

The data represent the average results from a range of global prices. In the PRU scenario, current hydrogen costs range between USD 3.5 and 6.5/kg using electricity prices of USD 50–200/MWh. By 2050, with USD 50–120/MWh for the electricity price, the hydrogen cost may decrease to USD 2.25–3.75/kg, resulting in an average reduction of more than 30%. In the ORE scenario, current hydrogen costs range between USD 2 and 4/kg using electricity prices of USD 30–150/MWh. By 2050, the hydrogen cost can decline to USD 0.7–1.3/kg with USD 20–80/MWh for the electricity price, an even more significant reduction (about 66% in hydrogen and nearly 45% for electricity price) than the PRU scenario. It is worth mentioning that the ORE scenario even assumes lower hydrogen and electricity price costs in 2020 than those in the PRU scenario. In detail, the hydrogen cost is 40% lower, and the electricity price is 28% lower in the ORE scenario. These significant reductions in hydrogen costs can lead to other cheaper hydrogen-based products, such as green renewable ammonia, whose price is estimated to drop from present USD 720–1400/ton to USD 310–610/ton by 2050, which is a drop of more than 50%.^78^^,^^82^

Moreover, hydrogen can achieve the most significant emission savings among all e-fuels, but only if the input electricity has a carbon intensity of no more than 160 g CO_2_/kWh. On the other hand, the life cycle GHG emissions of e-kerosene are more than hydrogen due to more production processes, which means its input electricity must have a carbon intensity of less than 110 g CO_2_/kWh to guarantee climate benefits.^83^

Figure 9C displays the estimated production of green hydrogen with renewable electricity consumption for 2030 and 2050 based on two scenarios (PRU and ORE, same as the definitions in Figure 9D). The amount of electricity consumed was represented by multiplying 0.01 for better formatting. In the PRU scenario (lower format), the production of green hydrogen is expected to increase from 5 Mt to 95 Mt per year by 2050. Meanwhile, the clean electricity used to produce this amount of hydrogen is projected to rise from 250 TWh to 5850 TWh annually. These data indicate that from 2030 to 2050, the capacity to produce hydrogen will likely increase by more than 18-fold, whereas there will be a more than 220% increase in electricity consumption. This implies hydrogen production by electrolysis is slightly less efficient in 2050 than in 2030. This could be due to the cautious assumptions made in this scenario. However, in the ORE scenario, hydrogen production is expected to rise from 9 to 160 Mt per year by 2050, which is a growth of about 17 times, whereas the related electricity consumption is projected to increase by less than 200%. It is also significant that hydrogen production in the ORE scenario is expected to be over 68% larger by the middle of this century than PRU’s. In general, it can be inferred that by 2050, more hydrogen can be produced from clean electricity with better efficiency. However, this estimation must be based on confident and optimistic assumptions about the advancement in electricity-based technology in the next few decades.^26^^,^^45^ The US Department of Energy’s (DOE’s) Energy Earthshots Initiative is the first of the six Energy Earthshots.^84^

It seeks to reduce the cost of clean hydrogen by 80% to $1 per 1 kg in one decade. Hydrogen is a clean and versatile energy carrier that can power various applications, including transportation, electricity generation, and industrial processes. However, the current cost of producing clean hydrogen is too high to make it a commercially viable option. If this can be achieved, the cost of electrolysis can be reduced by using more efficient catalysts and developing new membrane technologies. Thermochemical processes use heat to split water molecules into hydrogen and oxygen. These processes are more energy-intensive than electrolysis, but they have the potential to be more cost-effective in some applications. Reducing the cost of hydrogen production can significantly contribute to developing a clean energy economy.

## The total CAPEX and OPEX costs for DAC-e-fuel production

This section presents the total estimated costs for CAPEX, OPEX, and fuel costs until 2050 using low- and high-temperature DAC for carbon sources. The study’s financial and technical assumptions are based on market development and insights from the scientific literature.^26^^,^^35^^,^^63^

**Figure 10:**
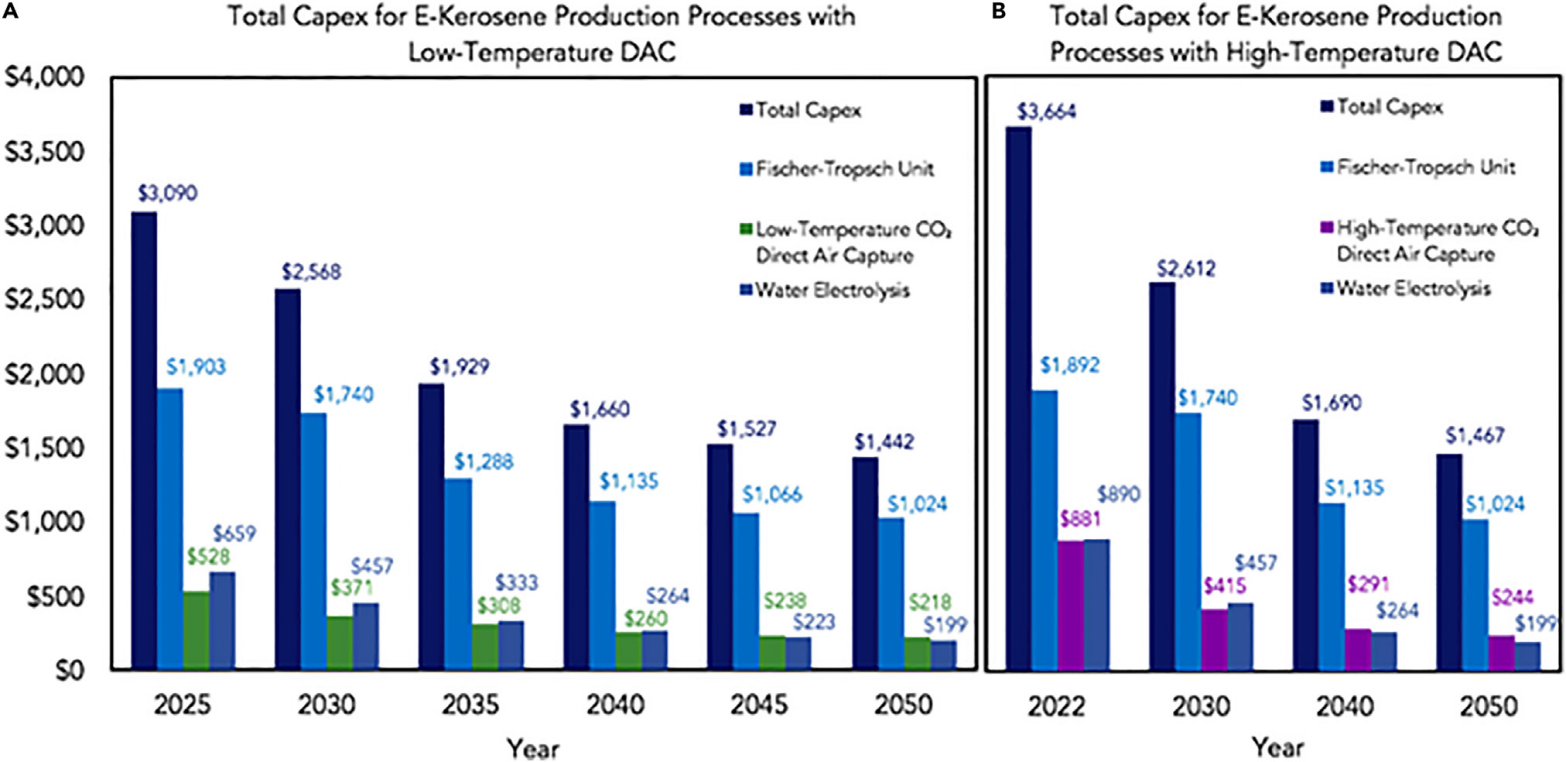
The projected total Capex for e-kerosene production processes until 2050 (A) Low-temperature DAC process. (B) High-temperature DAC process.

Figures 10A and 10B present financial and technical predictions for all generation technologies and show the decreasing trend in total costs over the years. The predictions are made at five-year intervals between 2020 and 2050.

## Investments and policies in the production of aviation e-fuels

This section focuses on the financial and policy aspects of producing aviation e-fuels, which are vital for achieving net-zero emissions in aviation. The financial and policy frameworks are underpinning the development of SAFs, DAC, green production, FT technology, and renewable energy. This includes the current costs, projected reduction, and the roles of different countries in providing support and policy incentives.

### Investment for producing aviation e-fuels

Investing in the production of SAF is crucial in achieving net-zero aviation emissions. Although the cost of producing SAF is higher than that of fossil fuels, the report predicts that the costs will decrease in the coming years. Countries such as Germany, France, and the Netherlands have provided financial support to promote the production of SAFs. The dena report highlights that Germany has launched an aviation fuels research program and provides funding for producing e-fuels. Additionally, the European Union has set a target for low-emission fuels to comprise 63% of aviation fuel by 2050, including e-fuels.^26^

### Investment for DAC

DAC is a vital technology for reducing carbon emissions from the aviation sector. However, the technology is still in its early stages, and substantial investment is required to improve it and make it more cost-effective. Countries like the United States, Canada, and the UK have already invested in developing DAC technology. According to the MDPI paper, DAC technology is expensive, and significant investment is needed to reduce its cost and scale up the technology. The paper recommends that public funding and policies support the deployment of DAC technology.^26^

Cost is one of the biggest obstacles stopping DAC from being commercially successful. Currently, CCUS plants are designed to capture around 90% of the CO_2_ from flue gas. Increasing capture rates beyond 90% increases costs due to higher energy and larger equipment requirements. The costs for CO_2_ sourced from DAC are currently higher than for CCU. Due to this, most production plants planned or in operation use CCU to obtain CO_2_ as a feedstock. Predictions suggest that the costs of DAC will vary between USD 340 and 540/tCO_2_, and it is also predicted to fall below USD 100/tCO_2_ by 2050.

According to the IEA’s 2022 report, if the waste heat from other processes (such as from the FT process) is used as process heat, costs can be reduced even further to approximately USD 50/tCO_2_ for S-DAC by 2050.^85^ This suggests that using heat from alternative sources such as waste heat from other processes and using renewable energy will reduce the cost significantly. Hence, developing DAC systems integrated with renewable energy systems such as wind and solar energy is paramount.^81^

L-DAC requires high temperatures that are still supplied by the combustion of natural gas, releasing CO_2_. This CO_2_ can be directly captured within the process to avoid being emitted into the atmosphere. However, this lowers the efficiency of CO_2_ capture (the ratio of emitted to captured CO_2_). Therefore, S-DAC technology, which requires low temperatures, is preferred. According to the IEA’s 2022 report, the costs for S-DAC are currently 1.5 times higher than for L-DAC.^85^ To reduce costs, S-DAC is better, as the waste heat from other processes can be utilized. This can reduce costs and enhance system integration if the processes are combined. Low-temperature heat can also be supplied using heat pumps, which can be powered using renewable sources of electricity such as biomass energy (e.g., BECCS).^81^

The major cost parameters include the sorbent working capacity, sorbent lifetime, cycle time, the required vacuum pressure, and the desorption temperature.

• The working capacity represents how much CO_2_ the sorbent can uptake per unit and depends on both the absorption and desorption capacity of the sorbent material. Higher working capacity is better, as this would mean less sorbent material is required, reducing costs.
• The sorbent lifetime refers to the time it takes for the sorbent uptake to drop below acceptable standards. The acceptable standards can vary from sorbent to sorbent and may depend on the working capacity of the sorbent. This impacts the costs associated with repeated sorbent purchasing.
• The cycle time helps us know how long it takes to undergo a complete capture and regeneration cycle. The longer the cycle time, the fewer cycles each sorbent undergoes yearly, driving up the amount of sorbent needed to achieve the same yearly capture. Hence, lesser cycle times are preferred.
• The desorption temperature impacts the thermal energy required by the system to achieve the optimum temperature by the sorbent to evolve the captured CO_2_. Higher desorption temperature means more energy requirements, which drives up the costs. Additional parameters, such as the cost of the sorbent material, also impact the economic viability of the process.

Changing the aforementioned parameters can help reduce costs effectively and optimize the process.^65^

**Figure 11:**
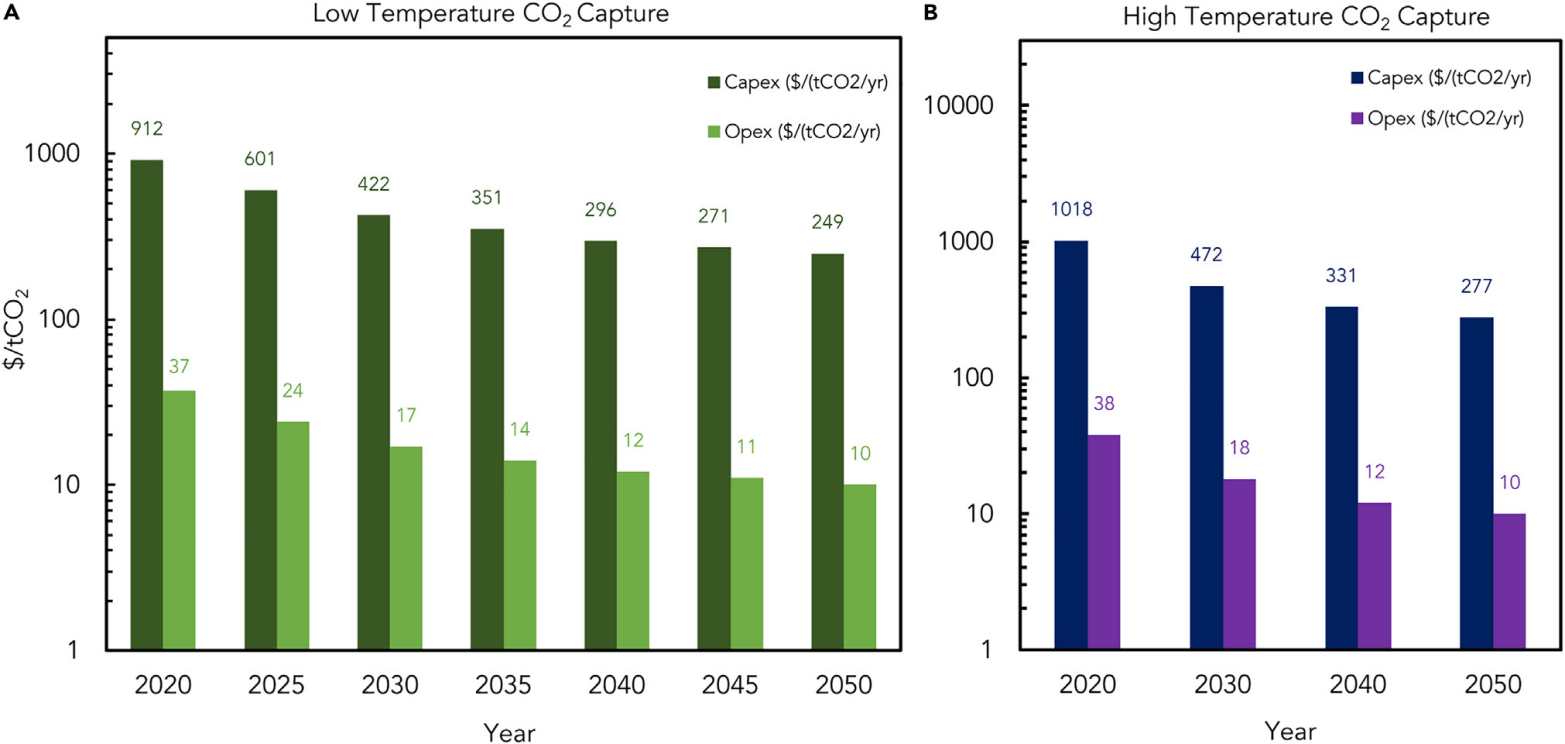
Projected Capex and Opex for DAC processes until 2050^26^^,^^35^^,^^67^ (A) Low-temperature DAC process. (B) High-temperature DAC process.

Figures 11A and 11B display the cost of S-DAC (low-temperature CO2 capture) and L-DAC (high-Temperature CO_2_ capture) per ton of CO_2_ for the next couple of years, respectively. It indicates that both processes' capital and operational costs reduce as the years progress. This will only be possible if the DAC systems are scaled up and their procedures are made more efficient by using efficient techniques such as reusing flue gases to heat the systems to the desired temperature. Cost acts as a hindering factor to the progress of DAC. But if the costs drop due to a massive influx of DAC CO_2_, the captured CO_2_ can be used to make cheap synthetic fuel, which can be used for any transportation system without any major modifications.

### Investment for green hydrogen production

The hydrogen aircraft is subject to uncertainty in the current aviation market due to technological barriers and the cost competitiveness of other low-emission aircraft. Among all factors, fuel costs tend to significantly impact the total cost of ownership (TCO).^86^ In modern aviation, most e-fuels rely on renewable hydrogen, accounting for 66%–83% of cost contribution.^87^^,^^88^ According to the climate targets in the Paris Agreement, global capacity for green hydrogen needs to grow 6,000- to 8,000-fold from 2021.^89^^,^^90^ Thus, renewable hydrogen will remain insufficient shortly. The good news is that renewable electricity costs are already competitive in many regions. According to the Inflation Reduction Act, the United States has announced a comprehensive subsidy program to stimulate the promotion of green hydrogen. Specifically, the price of green hydrogen may be reduced to USD 0.39/kg by 2030, much lower than the previous conventional prediction.^91^^,^^92^

To achieve carbon-neutral growth in 2030 (maintaining the same emissions levels as in 2019), an annual investment should reach about USD 40–50 billion in this decade. This number in 2050 would be about USD 175 billion. Of these investments, 94% are required for producing e-fuels, including about 35%–50% for the decline in the cost of renewable electricity. However, if this decline is slower than expected, biofuels may dominate the market if sufficient sustainable biomass is directed to the aviation sector. Overall, USD 5.1 trillion of total investments could be required by 2050 to deliver net zero in global aviation. To sum up, for future investment in aviation, when considering the use of green resources for decarbonizing, the priority of hydrogen should be higher than DAC-kerosene.^45^

Australia, Chile, and Saudi Arabia are some of the countries that hold great potential for the production of green hydrogen. However, significant investment is required to scale up the production of green hydrogen and reduce costs. The MDPI paper suggests that public policies such as carbon pricing and mandates for renewable energy can support the deployment of green hydrogen production.^45^^,^^93^

### Investment for FT

The FT technology has the potential to produce synthetic fuels that can help decarbonize the aviation sector. The report highlights that countries such as South Africa, Malaysia, and Qatar have already gained experience in using FT technology for this purpose. However, it also mentions that significant investment is required to scale up FT technology and reduce costs.^26^^,^^45^

### Investment for renewable energy (wind or solar)

As the dena report highlights, several countries, including Germany, Denmark, and Spain, have invested in wind and solar energy to produce renewable electricity. The report also notes that green hydrogen and power DAC technology can be produced using renewable energy. According to the McKinsey report, investment in renewable energy is crucial to achieving net-zero aviation emissions. The report suggests that the United States, China, and India have significant potential for renewable energy production, but significant investment is required to scale up production and reduce costs.^94^

Producing e-kerosene from renewable energy sources requires a significant increase in deploying renewable energy infrastructure such as wind turbines and solar panels. The cost of renewable electricity, the primary energy input for e-kerosene production, plays a crucial role in determining the overall cost competitiveness of e-kerosene.^65^^,^^95^^,^^96^^,^^97^

Significant investments in renewable energy infrastructure will be required to achieve renewable electricity production capacity. The report acknowledges that there are already significant investments in renewable energy projects in the European Union and the United States, and additional investments will likely be required to meet the future demand for renewable electricity.^47^^,^^81^^,^^98^

The report recognizes the importance of renewable energy for the production of e-kerosene and highlights that significant investment in renewable energy infrastructure will be required to achieve carbon-neutral aviation.^45^

Key policy measures in different countries for investment in producing aviation e-fuels, DAC, green H_2_ production, FT, and renewable energy are summarized in Tables 5 and 6.

**Table 5:** Investment in e-fuels, DAC, H_2_, FT, and renewable energy

Key policy measures for investment	USA	EU	China	India	Japan
Investment for aviation e-fuels	$5.5 billion (Biofuel producers tax credit)	Blending mandate (ReFuelEU Aviation)	$1.5 billion (National Biofuel Fund)	$10 million (Biofuel Purchase Program)	$0.26 billion (Biojet fuel production subsidy)
Investment for direct air capture (DAC)	$200 million (CarbonSAFE)	$410 million (Horizon Europe)	$259 million (National Key R&D Plan)	$70 million (National R&D Fund)	$48 million (Strategic Innovation Program for Energy Conservation Technologies)
Investment for green hydrogen production	$500 million (H2@Scale)	$1.8 billion (Fuel Cells and Hydrogen Joint Undertaking)	$15.7 billion (National Energy Administration)	$200 million (National Hydrogen Energy Mission)	$600 million (Green Innovation Fund)
Investment for Fischer-Tropsch (FT)	$1.2 billion (Advanced Fossil Energy Projects)	$5 billion (Innovation Fund)	$14.7 billion (National Energy Administration)	$300 million (National Bioenergy Mission)	$60 million (Green Innovation Fund)
Investment for renewable energy (wind or solar)	$35 billion (Production Tax Credit)	$46 billion (Investment Plan for Europe)	$83 billion (National Energy Administration)	$18 billion (National Solar Mission)	$23 billion (Feed-in Tariff Scheme)

Adapted from ref.^45^^,^^99^

**Table 6:** Policy measures for different energy sources in the following countries

Technologies	United States^a^	Germany	France	United Kingdom
Aviation E-fuels	Federal tax credits and renewable fuel standard 45Z, 45Q, and 45V∗∗	Subsidies and feed-in tariffs for renewable H_2_	Tax incentives for renewable fuels production	Incentives for the development of SAF
DAC	Tax credit and state-level policies	Renewable energy surcharge and tender system	Research funding and tax incentives	Tax incentives for carbon capture and storage
Green H_2_ production	Tax credit and research funding	Feed-in tariffs and grants	Subsidies and tax incentives for H_2_ production	Funding for research and development
FT	Federal tax credits and loan guarantees	Subsidies and grants for biofuels	Tax incentives for renewable fuels production	Research funding and tax incentives
Renewable energy	Production tax credits and investment tax credits	Feed-in tariffs and tax incentives	Feed-in tariffs and tax incentives	Contracts for differences and green investment bank

Adapted from ref.^98^^,^^100^^,^^101^^,^^102^

∗∗45Z Clean Fuel Production Credit: introduced by the Inflation Reduction Act 2022, this credit provides financial incentives for producing low-carbon transportation fuels, including sustainable aviation fuel (SAF), renewable diesel, and biodiesel. The credit amount varies based on the intensity of the fuel’s greenhouse gas (GHG) emissions. E-fuels, synthetic fuels produced from renewable electricity and carbon dioxide, could qualify for the 45Z credit depending on the intensity of their GHG emissions. However, the IRS is still finalizing the specific requirements for e-fuel eligibility.

∗∗45Q Carbon Capture and Sequestration Credit: this credit encourages investment in technologies that capture and permanently store carbon dioxide emissions from industrial facilities and power plants. The 45Q credit is not directly applicable to e-fuel production. However, some e-fuel production facilities may utilize carbon capture technologies, making them eligible for the 45Q credit on the captured carbon dioxide.

∗∗45V Clean Hydrogen Production Credit: this credit provides financial support for producing clean hydrogen, which can be used in various applications, including transportation and fuel cell power generation. Clean hydrogen can be used as a feedstock for e-fuel production. E-fuel facilities that utilize clean hydrogen produced at a qualified facility may be eligible for the 45V credit on the hydrogen used.

a In the United States, most of the tax credit is for biofuels and not for e-fuels, but the 45Z, 45Q, and 45V credits can be applied.

### Anticipating future enhancements

Several key improvements can be made to support deploying technologies for decarbonizing the aviation sector, including increasing investment in SAFs, green hydrogen production, and DAC technology. Developing supportive policies such as carbon pricing, renewable energy mandates, and financial incentives can also help deploy new technologies. Establishing international cooperation and coordination can accelerate the deployment of new technologies and the sharing of best practices. Public-private partnerships can also be developed to support the deployment of new technologies.

## Challenges and future prospects

E-fuels face several challenges that must be addressed before being widely adopted. One of the main issues is the high production cost, where the process of e-kerosene involves large amounts of energy and raw materials, which makes it complex and expensive. Currently, e-kerosene production costs around $8.80 per gallon in the United States, which is expected to decrease to $4 per gallon by 2050.^15^ However, this cost is still relatively high compared with fossil fuel costs. According to T&E and Ricardo Energy and Environment Research, the projected price of e-kerosene in 2030 will be between $144 and $250 per MWh, which is still two to three times the average price of fossil kerosene.^16^

Another challenge is the limited production capacity. The high cost and raw material requirements result in a small market size, and the current production capacity falls short of meeting the demand. Only around 8 Mt of SAF is produced yearly, projected to increase to 300–370 Mt with 1600–3400 plants by 2050.

The production of e-fuels relies on renewable energy sources like solar and wind power. However, geographical and climatic factors limit the supply of these sources, presenting a challenge to ensure a reliable and sufficient supply of green electricity. According to estimates, e-fuel production will require 535 TWh of renewable electricity by 2050, which makes up 22% of all renewable electricity demand in the transportation sector.^16^

Apart from these challenges, there are also technological hurdles to overcome. The current technology readiness level (TRL) for PtL processes, including e-fuel production, is relatively low, indicating the need for further technological advancements. Research and development are required to improve the efficiency of renewable electricity generation, electrolysis, and DAC technologies.

Addressing the challenges and promoting the prospects of e-kerosene requires both short-term and long-term actions.

*In the short term*, it is essential to establish stakeholder alliances and invest in infrastructure to support the industry’s deployment. Improving environmental models and data can optimize existing policies and inform the implementation of new ones. These actions can lay the foundation for supply chain development, maintenance, and continuous improvement.

*In the long term*, research and development efforts should focus on the scale and sustainability of raw materials, raw material logistics, and processing reliability. This will drive technological and strategic innovation. It is also crucial to reduce risks, accelerate fuel conversion technology, and provide financial support through creative financing and government loans to overcome project financing barriers. Consistent policy incentives and long-term deployment support are necessary. Fuel safety testing, evaluation, and regulation activities should be accelerated to streamline the approval process for new fuels.

This section examines the challenges and the prospects for key technologies crucial in decarbonizing the aviation sector. With current sorbents, DAC faces high costs, significant energy requirements, and low CO2 efficiency. Prospects include the development of efficient sorbents and the optimization of regeneration processes. Green hydrogen production faces challenges with cost, infrastructure, and the nature of renewable energy. FT technology grapples with high capital and operational costs and limited feedstock availability. Lastly, renewable energy investments, particularly in wind and solar, face challenges such as cost, intermittency, and grid integration, but ongoing technological advancements and grid enhancement efforts offer a positive outlook.

### Technology-specific hurdles and future prospects

This section examines the challenges and the prospects for key technologies crucial in decarbonizing the aviation sector. With current sorbents, DAC faces high costs, significant energy requirements, and low CO2 efficiency. Prospects include the development of efficient sorbents and the optimization of regeneration processes. Green hydrogen production faces challenges with cost, infrastructure, and the nature of renewable energy. The FT technology grapples with high capital and operational costs and limited feedstock availability. Lastly, renewable energy investments, particularly in wind and solar, face challenges such as cost, intermittency, and grid integration, but ongoing technological advancements and grid enhancement efforts offer a positive outlook.

#### Direct air capture

##### Challenges

DAC technology’s biggest challenge is its high cost. The initial investment required to build and operate DAC facilities is substantial, and the technology is still relatively new, meaning there are no economies of scale yet. In addition, DAC requires a significant amount of energy, so it is necessary to consider the availability and sustainability of power sources. Current DAC sorbents exhibit relatively low CO_2_ capture efficiency, requiring large amounts of energy for regeneration. The selectivity of sorbents toward CO_2_ is often limited, allowing other gases to be captured, reducing overall capture efficiency. Another challenge is developing scalable and efficient carbon capture materials and processes.^103^

##### Future prospects

New sorbents with enhanced CO_2_ capture efficiency and selectivity and reduce the energy requirements for regeneration, which are of great interest. Developing more efficient and cost-effective sorbents will reduce DAC implementation costs. Efforts are underway to optimize regeneration processes, reducing energy consumption and improving the overall efficiency of DAC systems.^104^ With advancements in technology and scaling, the cost of implementation is expected to decrease, making it more economical. Innovations in materials and processes can lead to more efficient and affordable carbon capture solutions. Moreover, as the need to address climate change becomes more urgent, there is potential for increased investment and policy support for DAC technologies.^26^

#### Green H_2_ production

##### Challenges

The production of green hydrogen faces several challenges, particularly related to cost and infrastructure. Green hydrogen production relies on renewable energy sources, such as wind or solar, which can be intermittent and geographically constrained. This poses challenges in ensuring a consistent and reliable supply of renewable energy for hydrogen production. Additionally, the cost of green hydrogen production is currently higher than fossil-fuel-based hydrogen, mainly due to the high capital costs of electrolysis equipment.

##### Future prospects

As renewable energy technologies continue to improve, they can help address the intermittent nature of renewable energy sources and make them more reliable. Improved forecasting models and scheduling algorithms can optimize green hydrogen production based on renewable energy availability, minimizing the impact of intermittency. Integrating energy storage solutions, such as batteries or pumped hydro storage, can store excess renewable energy during periods of high generation and utilize it for electrolysis when renewable energy is scarce. Enhancing grid flexibility and implementing demand-side management techniques can further balance supply and demand, ensuring a reliable electricity supply for green hydrogen production. Technological innovations and scaling up of production can also significantly lower the cost of electrolysis, making green hydrogen more affordable. Developing more efficient and cost-effective electrocatalysts can significantly reduce the energy requirements for electrolysis, lowering production costs. Enhancing the performance and durability of ion exchange membranes used in electrolysis can reduce maintenance costs and extend the lifespan of electrolysis cells. Furthermore, governments and private sectors worldwide increasingly recognize the potential of green hydrogen.^94^

#### Fischer-Tropsch technology

##### Challenges

Investing in FT technology can be challenging, mainly because of its high capital and operational costs. The FT process consists of complex and energy-intensive steps requiring substantial investments in large-scale facilities and infrastructure. Moreover, the availability of feedstocks like biomass or captured carbon can be limited and geographically specific, making it challenging to deploy this technology widely.

##### Future prospects

The advancements in process efficiency and catalyst development can help reduce FT facilities' capital and operational costs. Optimizing reactor designs, such as implementing multi-stage or fluidized bed reactors, can improve syngas conversion and product selectivity. Fine-tuning operating conditions, including temperature, pressure, and catalyst loading, can maximize product yield and minimize energy consumption. Implementing heat integration and waste heat recovery techniques can significantly reduce energy requirements and improve overall process efficiency. Developing advanced catalysts is pivotal in improving the FT process performance and cost-effectiveness. Developing catalysts with high activity can accelerate the FT reaction, increasing product yield and reducing reaction time. Tailoring catalysts to exhibit high selectivity toward desired products can minimize the formation of unwanted byproducts and improve the overall efficiency of the process. Developing catalysts with enhanced stability can extend their lifespan, reducing catalyst replacement costs and minimizing downtime. Additionally, with the increasing demand for sustainable and low-carbon fuels, there is an opportunity for policy support and investments in FT technology.^26^

#### Renewable energy (wind or solar)

##### Challenges

The investment in renewable energy, particularly wind and solar, is facing several challenges that include cost, intermittency, and grid integration. Although renewable energy costs have significantly reduced over the years, the upfront investment costs for large-scale wind or solar projects can still be substantial. Additionally, the intermittent nature of wind and solar energy sources poses a challenge to ensuring a stable and reliable power supply, which requires effective energy storage solutions. Integrating renewable energy sources into existing power grids also presents challenges, as grid infrastructure may need upgrades to accommodate the fluctuating nature of these sources. The expansion of renewable energy often necessitates significant investments in grid infrastructure to accommodate the increased power generation and transmission capacity. Also, placing renewable energy projects can be challenging due to land use concerns and potential environmental impacts, requiring careful planning and mitigation measures.

##### Future prospects

The prospects for investment in renewable energy, especially wind and solar, are promising despite the challenges. With the continuous advancements in technology, such as the development of more efficient solar panels and larger wind turbines, the costs of renewable energy systems can be further reduced while increasing their energy output.^45^

Continued research and development focused on improving energy storage technologies, such as batteries and pumped hydro storage, can address the intermittency issues. Investing in smart grid technologies and grid flexibility measures can enhance the integration of renewable energy sources into the grid.
